# Microbial Communities, Volatile Flavor Profiles and Metabolomic Characteristics During Traditional Hakka Huangjiu Fermentation

**DOI:** 10.3390/foods15060999

**Published:** 2026-03-11

**Authors:** Lin Cheng, Yujing Wang, Xin Feng, Bing Li, Yifang Chen, Feiliang Zhong, Xuegang Luo

**Affiliations:** Key Laboratory of Industrial Fermentation Microbiology of the Ministry of Education and Tianjin Key Laboratory of Industrial Microbiology, College of Biotechnology, Tianjin University of Science and Technology, Tianjin 300457, China; chl@mail.tust.edu.cn (L.C.); wangyujing_@mail.tust.edu.cn (Y.W.); fengxin1025@mail.tust.edu.cn (X.F.); li-bing@mail.tust.edu.cn (B.L.); chenyifang@mail.tust.edu.cn (Y.C.); flzhong91@tust.edu.cn (F.Z.)

**Keywords:** fermentation microecology, saccharification, post-fermentation, absolute quantitative sequence, PICRUSt, metabolic pathway, functional compound

## Abstract

The brewing of Traditional Hakka Huangjiu (THHJ) is usually divided into saccharification and post-fermentation. Microbial succession during saccharification is the major factor influencing the development of the volatile and non-volatile substances in THHJ during post-fermentation. This study systematically investigated the dynamic changes in microbial community, volatile substances and microbial metabolites by using absolute quantitative sequencing and multi-omics analysis. This study also reported that the correlation between microorganisms and substance biosynthesis was analyzed using PICRUSt. Absolute quantitative sequencing results showed that *Pediococcus*, *Saccharomycopsis*, *Rhizopus*, *Weissella*, and *Limosilactobacillus* were the dominant microbial genera during saccharification. 737 volatile compounds (170 esters, 94 hydrocarbons, 82 organoheterocyclic compounds) and 4370 metabolites (18 organic acids, 22 amino acids, 1124 peptides and 9 categories of functional compounds) were identified throughout the post-fermentation period. Sensory profiling revealed six main flavor attributes (Balsamic, sweet, rose, green, fruity, bitter) in THHJ and phenylethyl alcohol exerted the most prominent effect on the overall flavor of THHJ. Correlation analysis revealed that the biosynthesis of phenylethyl alcohol was potentially correlated with *Saccharomyces*, *Cyberlindnera*, *Pichia*, *Pediococcus*, *Pseudomonas* and *Lactococcus*. The biosynthesis of flavonoids was potentially correlated with *Pediococcus*, *Lactococcus*, and *Lactiplantibacillus*. These findings contribute to monitoring product quality and optimizing the processing techniques of THHJ.

## 1. Introduction

Traditional Hakka Huangjiu (THHJ) is a typical variety of Chinese rice wine (known as Chinese Huangjiu), an indigenous alcoholic beverage in China, renowned for its unique flavor, low alcohol content, and high nutritional value [1]. Chinese Huangjiu can be classified into three categories based on the type of fermentation starter known as “Jiu Qu,” which is made from different cereal materials: Yaoxiao Qu (main raw material: rice flour) Huangjiu, Mai Qu (wheat) Huangjiu and Hong Qu (red rice) [2] Huangjiu. The “Jiu Qu” utilized for THHJ belongs to the Yaoxiao Qu category, a fermentation starter composed mainly of rice flour supplemented with a small amount of Chinese herbal medicines, and dominated by microorganisms of the genera *Rhizopus* and Saccharomyces. Fungi have been confirmed to play a pivotal role in the saccharification process of THHJ by converting starch into fermentable sugars, which not only serve as carbon sources for microbial growth but also constitute the primary source of the wine’s sweetness [3]. Furthermore, enzymes secreted by microorganisms in the fermentation starter can hydrolyze proteins from glutinous rice (and occasionally indica rice) into peptides and amino acids, thereby providing essential nitrogen sources to support microbial growth and fermentation [4,5]. Although previous studies have initially elucidated some changes in microbial community structure during Chinese Huangjiu fermentation [1], the fermentation of THHJ is a synergistic process involving the combined actions of the entire microbial community, like other Chinese Huangjius; thus, analyzing only variations in the relative abundance of individual fungal or bacterial taxa possesses inherent limitations. Thus, further investigations are warranted to explore dynamic changes in microbial community structure during the fermentation of THHJ.

Although flavor metabolites and non-volatile metabolites (particularly bioactive compounds) in traditionally fermented beverages are highly complex, only a subset of volatile compounds contributes significantly to the overall flavor profile [6]. However, limited research has been conducted on this specific aspect in Chinese rice wine, and even fewer studies have focused on THHJ. Furthermore, THHJ fermentation comprises two distinct stages (saccharification and post-fermentation) [7]. During post-fermentation, microbial activity is suppressed, and changes in microbial community structure plateau, while volatile and non-volatile metabolites continue to evolve under the catalysis of residual enzymes. Notably, few studies have explored the correlations between microorganisms during saccharification and metabolite changes during post-fermentation. Therefore, a comprehensive understanding of the interactions among microorganisms during THHJ saccharification and their associations with alterations in volatile and non-volatile metabolites during post-fermentation is crucial for optimizing THHJ production processes, promoting the industrialization of this traditional food, and enhancing product quality.

In recent years, Chinese Huangjiu, including THHJ, has gained increasing recognition for its health-promoting properties. Beyond its palatable taste, THHJ serves as both a functional food to facilitate postnatal recovery for women and an auxiliary ingredient in various culinary preparations in parts of Guangdong Province, China. Its low alcohol content [8] enables volatilization during cooking, while retaining nutrients such as sugars and amino acids that are beneficial for postnatal recovery. Accumulating evidence has demonstrated that long-term and moderate consumption of THHJ or alcohol-free dishes prepared with it has been demonstrated to regulate blood pressure and glucolipid metabolism, as well as delay the aging process [9,10]. These health benefits are closely associated with its high content of bioactive compounds, including peptides, amino acids, vitamins, polysaccharides, flavonoids, and terpenoids [11]. These bioactive compounds exhibit considerable diversity in types and structural complexity. Although documented in previous studies, current research has been largely limited to the qualitative and quantitative analysis of structurally simple compounds. Notably, no studies have addressed the dynamic changes in bioactive compounds during THHJ production, nor have there been reports on their correlations with the microbial community. Advancements in detection technologies, however, now enable the accurate identification of complex compounds [12,13]. Coupled with various compound information databases [14,15], these technical advances lay a solid foundation for in-depth investigations. Consequently, systematic investigation of the dynamic changes in functional compounds during THHJ fermentation and their correlations with the microbial community will provide a theoretical basis for the development of functional THHJ products and the screening of probiotic strains.

Hence, the present study aimed to conduct integrated multi-omics analyses of THHJ, specifically focusing on characterizing the microbial community structure during saccharification as well as the volatile compoundomics and metabolomics during post-fermentation—addressing the existing gaps in understanding microbial synergy, dynamic metabolite changes, and their correlations in THHJ production. To achieve this, high-throughput sequencing of bacterial 16S rRNA genes and fungal ITS rRNA genes, headspace solid-phase microextraction-gas chromatography-mass spectrometry (HS-SPME-GC-MS), and liquid chromatography-mass spectrometry (LC-MS) were employed. Additionally, absolute quantitative analysis was utilized to evaluate the associations between bacterial and fungal genera and target metabolites, while volatile compoundomics and metabolomics data were annotated using the KEGG and HMDB databases to systematically explore metabolite classes that have not been previously reported in THHJ-related studies. Findings from this study are anticipated to provide a theoretical basis and technical support for reducing raw material waste and optimizing THHJ fermentation control, as well as data support for exploring the bioactive potential of THHJ and developing functional food products—fulfilling the research needs of clarifying microbial-metabolite interactions, elucidating dynamic changes in bioactive compounds, and promoting the industrialization and functional development of THHJ.

## 2. Materials and Methods

### 2.1. Chemicals and Reagents

Glutinous rice and indica rice were acquired from a local agricultural product dealer (Meizhou, China). The fermentation starters of “Jiu Qu” were obtained from Hakkasfood Co., Ltd. (Meizhou, China). Other chemicals were of analytical grade and obtained from Chemical Reagent Factory (Guangzhou, China).

### 2.2. Preparation of THHJ and Samples

The THHJ was prepared according to conventional production techniques. Briefly, 1000 g of glutinous rice was washed and soaked in water at 20–25 °C for 24 h, and then steam-cooked to starch gelatinization. The cooked glutinous rice was cooled to 25 °C and mixed with 10 g “Jiu Qu” (addition ratio 1%). Afterward, the mixture was transferred to an open container and cultured at 25 ± 1 °C. After 96 h of cultural and saccharification process, 300 g of high-proof liquor (obtained through distillation of low-alcohol rice wine, approximately 50% vol) was added to the mixture, maintaining elevated alcohol conditions (20–25% vol) with subsequent post-fermentation maturation over 42 days (20 ± 1 °C). Samples were taken during the saccharification process (every 24 h) and post-fermentation process (every 7 d).

### 2.3. Determination of Physicochemical Indicators

The detection methods for the physicochemical indicators of THHJ shall be in strict accordance with the Chinese National Standard GB/T 13662-2018 Huangjiu [16]. Total sugar content was determined according to the standard. Briefly, Fehling solution I (5 mL) and II (5 mL) were added to a 250 mL triangular flask, and 50 mL of water and glucose standard solution were added. The mixture was heated to boiling and maintained for 2 min before adding two drops of methylene blue, indicating liquid and titrating with glucose standard solution within 1 min. The total volume of glucose standard solution consumed to reach a clear solution was recorded and used to calculate the total sugar content. The alcohol content was detected by using a hydrostatic gauge. Total acids were measured by titrating a sample with 0.1 mol/L NaOH. The pH value of the samples was detected by using a digital pH meter (MI150, Milwaukee, Szeged, Hungary).

### 2.4. DNA Sequencing and Bioinformatic Analysis

Saccharification samples were analyzed using Accu16S^TM^ (Accurate 16S absolute quantification sequencing) and AccuITS^TM^ (Accurate ITS absolute quantification sequencing) [17]. Total DNA was extracted using the FastDNA^®^ SPIN Kit for Soil (MP Biomedicals, Santa Ana, CA, USA) according to the manufacturer’s instructions. The integrity of genomic DNA was detected through agarose gel electrophoresis, and the concentration and purity of genomic DNA were detected through the Nanodrop 2000 and Qubit 3.0 Spectrophotometer (Thermo Fisher Scientific Inc., Waltham, MA, USA). Multiple spike-ins with identical conserved regions to natural 16S and ITS2 rRNA genes and variable regions replaced by random sequence with 40% GC content were artificially synthesized. Then, an appropriate proportion of spike-in mixture with known gradient copy numbers was added to the sample DNA. The V3-V4 hypervariable regions of the 16S rRNA gene and spike-ins were amplified with the primers 341F (5′-CCTACGGGNGGCWGCAG-3′) and 805R (5′-GACTACHVGGGTATCTAATCC-3′). The ITS2 hypervariable regions of the ITS2 rRNA gene and spike-ins were amplified with the primers 341F. The Illumina NovaSeq 6000 sequencer (Illumina, Inc., San Diego, CA, USA) was used for sequencing. The raw read sequences were processed in QIIME2 (Version 2025.1, Caporaso Lab, Northern Arizona University, Flagstaff, AZ, USA) [18]. The adaptor and primer sequences were trimmed using the cutadapt plugin. The DADA2 plugin was used for quality control and to identify amplicon sequence variants (ASVs) [19]. Taxonomic assignments of ASV representative sequences were performed with a confidence threshold of 0.8 by a pre-trained Naive Bayes classifier, which was trained on the Greengenes (version 13.8, Lawrence Berkeley National Laboratory, Berkeley, CA, USA). Then the spike-in sequences were identified, and reads were counted. A standard curve for each sample was generated based on the read-counts versus spike-in copy number, and the absolute copy number of each ASV in each sample was calculated by using the read-counts of the corresponding ASV. Since the spike-in sequence is not a component of the sample flora, the spike-in sequence needs to be removed in the subsequent analysis [20].

### 2.5. Volatile Organic Compounds Analysis

Post-fermentation samples were analyzed by using HS-SPME-GC-MS [21]. Standard solution preparation: n-Alkane standard solutions (C10–C29) were prepared by adding 770 μL of n-hexane into a 1.5 mL centrifuge tube, followed by sequential addition of appropriate amounts of commercial mixed standards (C10–C25) and individual n-alkane standards (C26, C27, C28, C29), and vortex mixing to obtain a stock solution at 50 μg/mL; this stock was then diluted to 10 μg/mL and analyzed alongside samples in the same batch. For sample pretreatment, 500 μL of sample (actual volume as used) was transferred into a 20 mL headspace vial, to which 2.5 μL of internal standards (naphthalene-d8: 20 μg/mL; n-pentadecane-d32: 50 μg/mL) and 4 mL of saturated sodium chloride aqueous solution were added, and the vial was immediately sealed before analysis under the specified instrument conditions. Analyses were performed using a Thermo Orbitrap Exploris GC gas chromatography-mass spectrometry (GC-MS) system (Thermo Fisher Scientific Inc., Waltham, MA, USA) with headspace solid-phase microextraction (HS-SPME) as follows: a SPME Arrow Fiber (120 μm, DVB/Carbon WR/PDMS, Thermo Fisher Scientific Inc., Waltham, MA, USA) was employed under constant incubation mode, with incubation and extraction temperature at 60 °C, incubation agitation speed at 500 rpm, incubation time of 20 min, extraction stirrer speed at 200 rpm, extraction time of 10 min, inlet temperature at 250 °C, desorption time of 5 min, fiber conditioning pre- and post-desorption times of 2 min each, fiber conditioning temperature at 260 °C, and total analysis time of 25 min. Chromatographic separation was achieved on a VF-WAXms capillary column (25 m × 0.25 mm × 0.2 μm, Agilent CP9204, Agilent Technologies, Inc. Santa Clara, CA, USA) with split injection (split ratio 10:1); the inlet temperature was 240 °C, high-purity nitrogen was used as carrier gas at 1.0 mL/min, septum purge flow rate was 3 mL/min, and the temperature program was: initial 40 °C (held for 0 min), ramped to 120 °C at 8 °C/min, then to 230 °C at 20 °C/min (held for 4.5 min), with a total run time of 20 min. Mass spectrometric conditions included full-scan mode (m/z 35–500), Orbitrap resolution of 30,000, standard AGC target, electron impact (EI) ionization, ion source temperature at 250 °C, repeller voltage of 10 V, source offset of 5 V, lens 1 at −50 V, lens 2 at −0.5 V, lens 3 at −35 V, electron lens at 15 V, electron energy of 70 eV, and emission current of 50 μA. For quality control (QC), three QC samples were prepared by mixing all test samples, processed identically to actual samples, and injected randomly into the sequence; their repeatability was used to assess instrumental stability, identify high-variability variables, and ensure result reliability.

The raw data files acquired from GC/MS OE were subjected to library searching, metabolite identification, and data preprocessing using Thermo^TM^ Compound Discoverer^TM^ Software (version 3.3.SP3, Thermo Fisher Scientific Inc., Waltham, MA, USA) [22]. Specifically, the software initially matched the acquired mass spectral data against reference metabolic databases, and metabolites were annotated based on the mass spectral matching degree. The databases employed for spectral matching included NIST-2023 (https://www.nist.gov/srd/nist-standard-reference-database-1a accessed on 20 January 2026) and GC-orbitrap flavor and fragrances (https://documents.thermofisher.cn/TFS-Assets/MSD/Specification-Sheets/flavors-and-fragrances-en.pdf accessed on 20 January 2026). After annotation, a three-dimensional data matrix (xlsx format) was exported, which contained information on sample names, metabolite identities, and peak areas. Subsequently, false-positive peaks (including background noise, column bleed, and derivatization reagent peaks) were excluded from the data matrix, followed by data deduplication and peak alignment procedures to ensure data quality.

### 2.6. Metabolomics Determination

Post-fermentation samples were analyzed by using LC-MS [23]. Exactly 100 ± 5 mg of each sample was weighed into a 2 mL centrifuge tube, followed by the addition of one 6 mm diameter grinding bead. Then, 800 μL of extraction solution (methanol: water = 4:1, v:v) containing four internal standards (including L-2-chlorophenylalanine at 0.02 mg/mL) was added to the tube. The samples were ground using a cryogenic tissue grinder for 6 min at −10 °C and 50 Hz, and subsequently subjected to low-temperature ultrasonic extraction for 30 min at 5 °C and 40 KHz. After extraction, the samples were incubated at −20 °C for 30 min, then centrifuged at 13,000× *g* and 4 °C for 15 min. The supernatant was transferred to injection vials with inner liners for instrumental analysis. Additionally, 20 μL of supernatant from each sample was pooled to prepare quality control (QC) samples. The LC-MS analysis was performed on a Thermo Fisher Scientific UHPLC-Orbitrap Exactive system (Thermo Fisher Scientific Inc., Waltham, MA, USA). For the C18 column separation: An ACQUITY UPLC BEH C18 column (100 mm × 2.1 mm i.d., 1.7 μm; Waters, Milford, MA, USA) was used. The mobile phase consisted of solvent A (2% acetonitrile in water, containing 0.1% formic acid) and solvent B (acetonitrile, containing 0.1% formic acid). The injection volume was 3 μL, and the column temperature was maintained at 40 °C. For the Amide column separation (optional): An ACQUITY UPLC BEH Amide column (100 mm × 2.1 mm i.d., 1.7 μm; Waters, Milford, MA, USA) was employed. The mobile phase comprised solvent A (95% acetonitrile in water, containing 5 mM ammonium acetate) and solvent B (5% acetonitrile in water, containing 10 mM ammonium acetate). The injection volume was 3 μL, with the column temperature set at 40 °C. Mass spectrometric detection was conducted using electrospray ionization (ESI) in both positive and negative ion modes. The detailed parameters were as follows: scan range, 70–1050 *m*/*z*; sheath gas flow rate, 50 arb; aux gas flow rate, 13 arb; heater temperature, 450 °C; capillary temperature, 320 °C; spray voltage, 3500 V (positive mode) and −3000 V (negative mode); S-Lens RF Level, 40; normalized collision energy, 20, 40, and 60 eV; resolution for full MS, 70,000; resolution for MS^2^, 17,500.

After instrumental analysis of the samples, the obtained raw data were analyzed with reference to previous studies [24,25]. The raw data were subjected to peak detection, extraction, alignment, and integration using Progenesis QI (Waters Corporation, Milford, MA, USA). Metabolite annotation was achieved through the integration of three databases: the Human Metabolome Database (HMDB, http://www.hmdb.ca/ accessed on 20 January 2026) and METLIN (https://metlin.scripps.edu/ accessed on 20 January 2026). To eliminate or mitigate errors incurred during experimental and analytical procedures, qualitative data preprocessing was performed as follows: first, features with more than 20% missing values within each group were excluded from the raw data; subsequently, the remaining missing values were imputed using the minimum value across all samples; finally, the response intensities of the sample mass spectral peaks were normalized via total sum normalization to obtain a normalized data matrix. Furthermore, variables with a relative standard deviation (RSD) > 30% in quality control (QC) samples were discarded, and the resulting data were subjected to log10 transformation to generate the final data matrix for subsequent analyses. Statistical analyses, including principal component analysis (PCA) and orthogonal partial least squares-discriminant analysis (OPLS-DA), were conducted using the ropls package (Version 1.6.2) in R Studio (Version 2026.01.0+392). Metabolite annotation was further complemented using the HMDB and Kyoto Encyclopedia of Genes and Genomes (KEGG) database (https://www.kegg.jp/kegg/pathway.html accessed on 20 January 2026), whereas pathway enrichment analysis was performed using the scipy.stats (Version 1.17.0) package in Python (Version 3.13.1).

### 2.7. Statistical Analyses

All data were expressed as mean ± SD of three independent experiments. Significant differences among different groups were compared by one-way analysis of variance (ANOVA) with SPSS 18.0 software (SPSS Inc., Chicago, IL, USA). A significant difference was considered at the level of *p* < 0.05.

## 3. Results and Discussion

### 3.1. Dynamics Changes in Physicochemical Parameters During Saccharification and Post-Fermentation Process

**Figure 1:**
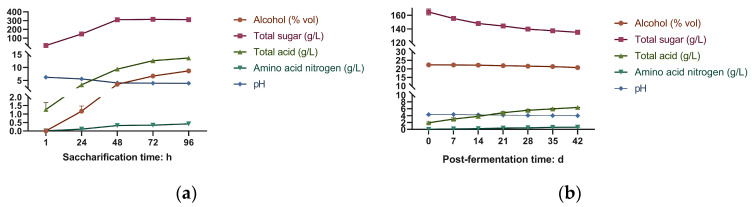
Physicochemical parameters during saccharification (**a**) and post-fermentation process (**b**).

Physicochemical parameters are critical for monitoring the fermentation process of THHJ. As widely recognized, glucose, sucrose, and maltose serve as the three major fermentable sugars in Huangjiu fermentation, primarily derived from the degradation of starch in rice/wheat by α-amylase and glucosidase from “Jiu Qu” [26]. During the saccharification of THHJ (Figure 1a), the total sugar content increased significantly from 15.04 g/L to 310.86 g/L (*p* < 0.05), while the alcohol content rose from 0% (*v*/*v*) to 8.67% vol (*p* < 0.05). Concurrently, the total acid content increased from 1.27 g/L to 13.65 g/L (*p* < 0.05), and the amino nitrogen content increased from 0.01 g/L to 0.45 g/L (*p* < 0.05). In contrast, the pH value decreased slightly from 6.23 to 3.89 (0.05 < *p* < 0.10). These parameters exhibited significant changes during the initial 1–48 h of saccharification and stabilized between 72 and 96 h, at which point saccharification was deemed complete; extending this period was deemed unnecessary for process efficiency. Following the addition of high-proof rice wine to elevate the system’s alcohol content to over 20% vol, the post-fermentation process was initiated. At the onset of post-fermentation, the initial physicochemical profile of THHJ was as follows: total sugar content (164.81 g/L), alcohol content (22.3% vol), total acid content (1.93 g/L), amino nitrogen content (0.01 g/L), and pH (4.33). During post-fermentation, low-molecular-weight sugars were predominantly consumed by microorganisms or enzymatic hydrolysis, contributing to the accumulation of organic acids. Meanwhile, proteases remained active, hydrolyzing macromolecular proteins into peptides and amino acids. After 42 days of post-fermentation (Figure 1b), the total sugar content decreased significantly to 135.27 g/L (*p* < 0.05), while the alcohol content slightly declined to 20.7% vol. Conversely, the total acid content increased significantly to 6.38 g/L (*p* < 0.05), and the amino nitrogen content rose to 0.64 g/L (*p* < 0.05). The pH value marginally decreased to 3.99, with no significant change attributed to the complex matrix of THHJ, which may have masked the effect of free acids, indicating that pH is not a critical parameter for THHJ quality assessment. Overall, the post-fermentation process of THHJ proceeded moderately with no drastic changes in physicochemical parameters. This observation suggests that microbial activity was strongly inhibited under high alcohol conditions, although microorganisms still metabolized sugars for survival. The gradual changes in physicochemical indices also reflect the suppression of enzymatic activity by high alcohol concentrations. Technologically, the post-fermentation of THHJ is typically terminated when physicochemical parameters stabilize, allowing for subsequent operations such as pressing for wine extraction and sterilization. However, metabolic products, including volatile flavor compounds, continue to evolve even after the stabilization of physicochemical indices. Further investigations into the mechanisms governing the dynamic changes in volatile flavor compounds during fermentation are warranted, as this will facilitate the optimization of processing conditions and the improvement of THHJ quality in future studies.

### 3.2. Dynamics Changes in Microbial Community During Saccharification Process

**Figure 2:**
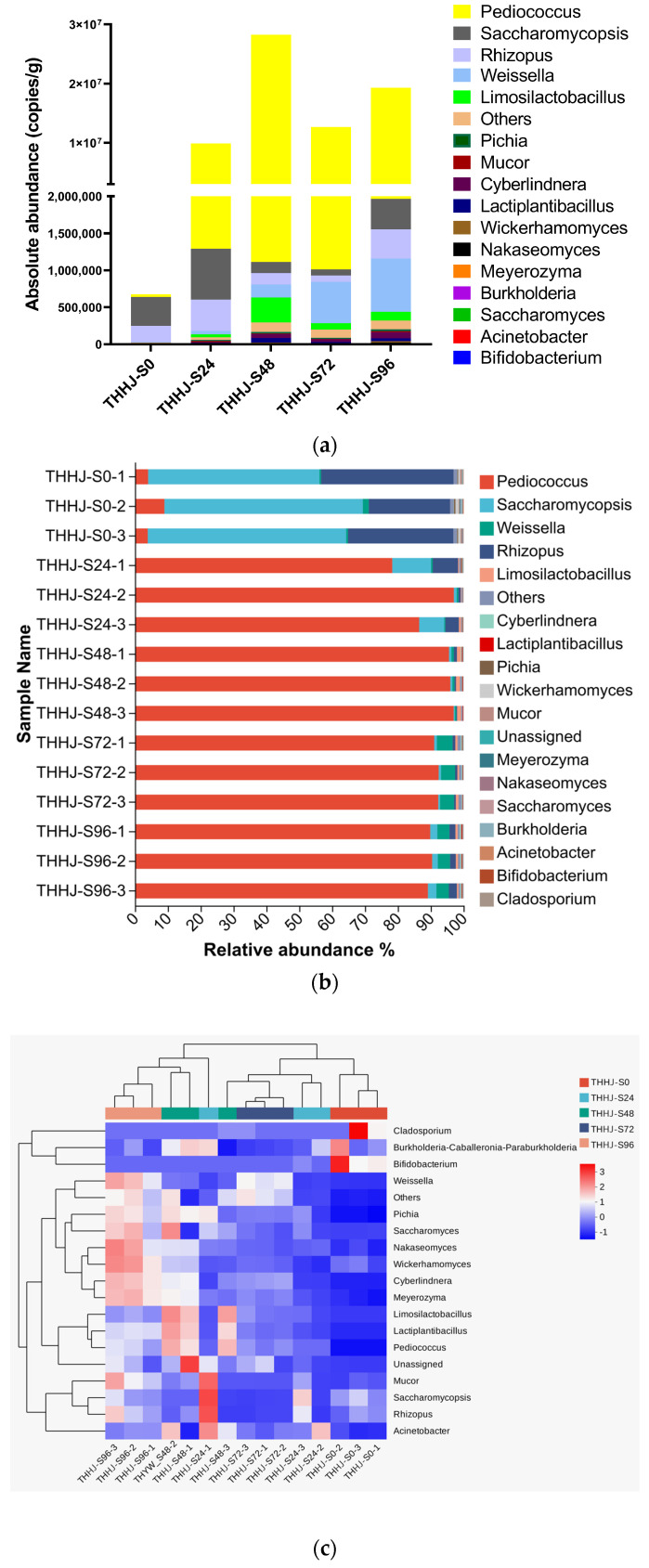
Absolute abundance of the microbial genera during the saccharification process of THHJ (**a**), relative abundance of the microbial genera (**b**), heatmap and dendrogram of the abundant (**c**).

Studies have demonstrated that microorganisms play a crucial role in the production of THHJ, particularly through the synthesis of various metabolites that contribute to its flavor and taste [3,27,28]. The microbial community composition obtained by traditional sequencing analysis methods is often presented based on the relative abundance of each microorganism in the community. This result can intuitively reflect the dynamic changes in the microbial structure in the fermentation system; however, such a relative abundance-based presentation cannot provide specific quantitative information. It also fails to break the analytical barrier between bacteria and fungi, precluding their integrated analysis. Therefore, this study employed absolute quantitative sequencing to quantitatively determine the microbial gene copy numbers during the saccharification process of THHJ, and conducted an integrated analysis of the composition of the two major microbial groups (bacteria and fungi) using the gene copy number per gram of sample as the unit. As shown in Figure 2, at the beginning of the saccharification process (0 h), only a small amount of bacteria was detected in the system, while a large number of gene copies of *Saccharomycopsis* (392,793 copies/g) and *Rhizopus* (218,184 copies/g) were identified. This indicates that both were the main microorganisms in THHJ Jiuqu (the fermentation starter). Subsequently, during 0–24 h of saccharification, various microorganisms in the system began to grow, reproduce, and exert their metabolic activities. During this period, the proliferation rate of bacteria was much faster than that of fungi, and aerobic or facultative anaerobic bacteria such as *Pediococcus* (up to 8,578,489 copies/g), together with *Saccharomycopsis* (688,269 copies/g) and *Rhizopus* (425,341 copies/g), occupied a dominant position. During 24–48 h of saccharification, the gene copy numbers of *Saccharomycopsis* (688,269 copies/g) and *Rhizopus* (425,341 copies/g) remained relatively stable, followed by a significant decrease in *Pediococcus* (from 27,145,684 to 11,623,550 copies/g) and *Limosilactobacillus* (from 336,958 to 85,077 copies/g) during 48–72 h. This reduction may be attributed to the accumulation of ethanol in the system, which inhibits microbial proliferation. During 72–96 h of saccharification, the gene copy numbers of *Saccharomycopsis* (up to 84,323 copies/g) and *Rhizopus* (up to 393,739 copies/g) rebounded. This phenomenon may be explained by the production of organic acids by microorganisms during saccharification, which led to a decrease in the environmental pH. At 72 h of saccharification, the pH dropped to 3.5, which is below the optimal growth pH range of *Pediococcus*, while acidophilic microorganisms such as *Rhizopus* and *Saccharomycopsis* could still grow normally at this pH. Notably, during 72–96 h of saccharification, the gene copy number of *Pediococcus* also rebounded (up to 17,292,765 copies/g), reflecting that the growth of *Pediococcus* during saccharification is dependent on *Rhizopus* and *Saccharomycopsis*. This dependency may be due to the enzymes produced by *Rhizopus* and *Saccharomycopsis* that decompose biological macromolecules into nutrients available for *Pediococcus* growth. Furthermore, the gene copy number of *Weissella* continuously increased throughout the saccharification process (from 6184 to 721,569 copies/g), and this microorganism was identified as *Weissella_paramesenteroides*. This species has been detected in “Jiu Qu” of many Chinese fermented wines as well as other fermented foods (e.g., kimchi and yogurt). It was a microaerophilic, lactic acid-producing microorganism with complex nutritional requirements. Its massive growth consumes a large amount of micronutrients in addition to carbon and nitrogen sources, which may be another factor contributing to the temporary reduction in *Pediococcus* abundance. Comprehensive analysis revealed that the dominant microorganisms during the saccharification process were *Pediococcus*, *Saccharomycopsis*, *Rhizopus*, and *Weissella*, and certain interactions exist among these microorganisms, which will be further investigated in future studies. At the end of saccharification, the sugar content and alcohol concentration in the fermentation system tend to reach a relatively balanced state. At this point, the addition of alcohol or water to the fermentation system determines the type of THHJ. When water is added for post-fermentation, the fermentation system is in an anaerobic state, and anaerobic or facultative anaerobic microorganisms such as *Pediococcus*, *Limosilactobacillus*, and *Saccharomycopsis* continue to exert their metabolic activities, consuming sugars and nutrients to produce ethanol, flavor compounds, and other metabolites, which is known as dry-type THHJ [29]. When ethanol is added to the saccharified mixture, the fermentation system is in a state where the high ethanol concentration inhibits microbial activity, allowing most of the sugars to be retained, and this is the source of the sweetness of sweet-type THHJ. However, extracellular enzymes produced by microorganisms, such as glucoamylase and protease, will continue to catalyze the raw materials to form monosaccharides, polysaccharides, polyols, peptides, and amino acids.

### 3.3. Volatile Flavor Compoundomics Changes During Post-Fermentation Process

#### 3.3.1. Statistical Difference in Volatile Flavor Compounds

**Figure 3:**
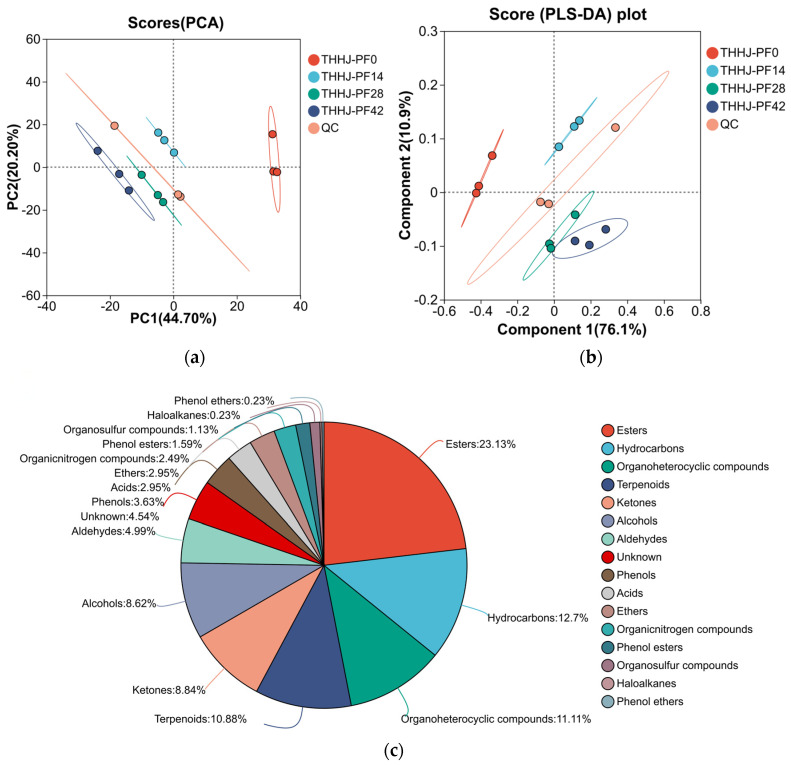
Principal component analysis score plot (**a**); Partial least squares-discriminant analysis score plot (**b**); Pie chart of volatile compound classification (top 16 categories sorted by quantity) (**c**).

PCA score plot (Figure 3a) showed that Principal Component 1 (PC1) and Principal Component 2 (PC2) explained 44.70% and 20.20% of the total variance, respectively. Partial Least Squares Discriminant Analysis (PLS-DA) revealed that Component 1 and Component 2 accounted for 76.1% and 10.9% of the variance (Figure 3b), respectively. As clearly observed in the PLS-DA score plot, samples at the initial stage of post-fermentation (THHJ_PF0, 0 days) were distinctly separated from those at the other three time points, while samples from the late post-fermentation stage (28–42 days) clustered closely together. This observation indicates that the major changes in volatile flavor compounds (VFCs) occurred during the early post-fermentation period (0–14 days), with the profiles tending to stabilize in the late stage. A total of 737 VFCs were detected and identified during the post-fermentation process (Spreadsheet S1), and chemotaxonomic annotation showed that esters were the most abundant class, with 180 compounds accounting for 24.42% of the total VFCs (Figure 3c). Additionally, OPLS-DA was performed to screen for significantly changed VFCs based on the criteria of variable importance in projection (VIP > 1 and *p* < 0.05). The dynamic changes in these differential VFCs are illustrated in Figure S1 (highlighting the top 5 metabolites by FC value). Among these, phenylethyl alcohol in THHJ_PF42 (42 days of post-fermentation) exhibited the highest VIP value (VIP = 16.284) with significant upregulation. 3,5,5-Trimethyl-2-cyclopenten-1-one showed the most significant statistical difference (*p* = 7.48 × 10^7^ upregulated). (2Z)-2-Phenyl-2-butenal had the maximum FC value of 394.6069, representing the highest fold increase, while 4-isopropyl-5,5-dimethyl-5H-furan-2-one displayed the most pronounced downregulation with an FC value of 0.0444. Phenylethyl alcohol, a common flavor compound in Chinese rice wine and a key aroma-active substance in THHJ [30], possesses multiple sensory characteristics, such as fruity and rose-like aromas. 3,5,5-Trimethyl-2-cyclopenten-1-one, a flavor compound derived from rice, contributes complex aromas, including nutty and roasty notes [31]. (2Z)-2-Phenyl-2-butenal, characterized by sweet and fruity aromas, is classified as an allowed food additive by the Chinese national standard GB 2760-2007 [32] and is widely used in formulating flavors for cocoa, honey, and coffee. Beyond these key compounds, the VFCs in THHJ also included various alcohols, aldehydes, acids, and other classes (Figure 3c), which collectively contribute to the unique sensory profile of THHJ. Current studies indicate that the types and contents of flavor compounds in Huangjiu are profoundly influenced by three key factors: raw materials [33], microbial communities in the starter [34], and production processes [35]. To maintain the traditional characteristics of Huangjiu products, it is necessary to follow corresponding practices in these three aspects.

#### 3.3.2. Sensory Flavor Characteristics

**Figure 4:**
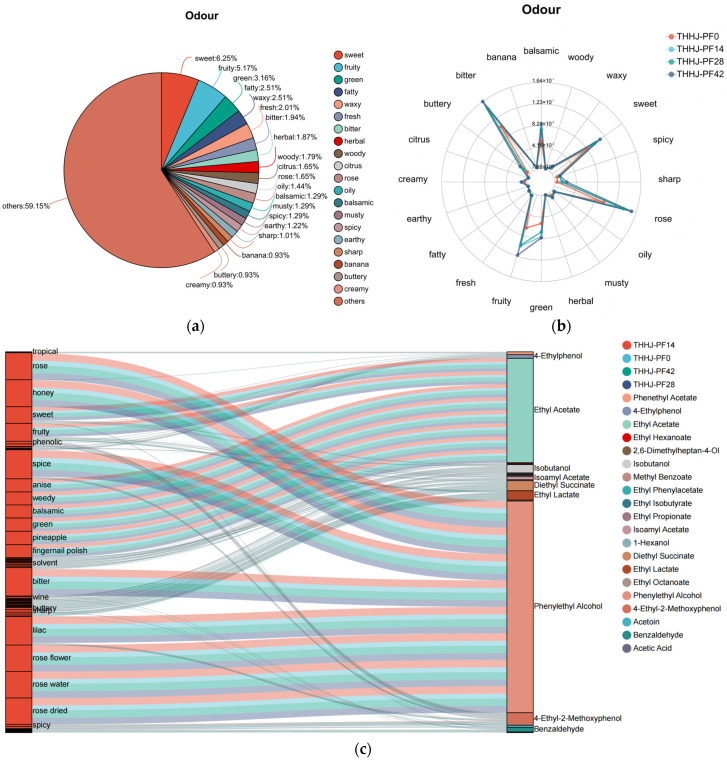
Classification diagram of volatile compounds (top 20 categories sorted by quantity) (**a**); Relative content of different compound types (**b**); Sankey diagram of sensory flavor characteristics and flavor compounds (**c**).

Sensory flavor profile analysis is widely utilized for evaluating the flavor quality of Chinese rice wine, with numerous relevant studies reported previously [36]. Conventionally, such sensory evaluation is performed by trained panelists, in conjunction with instrumental analyses using an electronic nose (e-nose) and an electronic tongue (e-tongue). In the present study, the sensory flavor profile of THHJ was analyzed based on the previously determined relative abundances of VFCs, followed by comprehensive classification according to their characteristic flavor attributes. The sensory flavor profile of total VFCs during the post-fermentation of THHJ is illustrated in Figure 4a. A total of 20 distinct flavor categories were identified for the ch/aracterized VFCs, among which more than 50% remained unclassified. Among the classified VFCs, 6.25% exhibited a sweet flavor, 5.17% a fruity flavor, and 3.61% a green flavor. Additionally, Figure 4b depicts the temporal evolution of sensory flavor intensities at 0, 14, 28, and 42 days of post-fermentation. It can be clearly observed that the VFCs of THHJ collectively contribute to its characteristic flavor, including bitter, fruity, green, rose, sweet, and balsamic notes. After 42 days of post-fermentation, changes in the VFC composition resulted in a significant enhancement (*p* < 0.05) of these flavor intensities. Notably, as a single VFC can possess multiple flavor attributes, each specific flavor in the Huangjiu is collectively contributed by multiple compounds [37]. The overall sensory flavor of THHJ is dominated by a small number of key VFCs. Figure 4c presents the top 20 VFCs in THHJ ranked by relative abundance, among which phenylethyl alcohol (relative abundance: 4.24) and ethyl acetate (2.08) were the most abundant. Other compounds, such as ethyl lactate and isoamyl acetate, contributed less to the flavor than phenylethyl alcohol and ethyl acetate, but these compounds are indispensable, as they synergistically shape the characteristic flavor of THHJ. Nevertheless, this study only summarized the flavor characteristics of THHJ based on the relative contents and flavor attributes of the substances. Although this method was adopted, certain limitations still exist. In fact, flavor characteristics are difficult to quantify. Current relevant studies usually employ the relative odor activity value (ROAV) to identify key flavor compounds [38]. The findings from this part of the study can provide a general direction for determining the key flavor compounds in THHJ and provide a general sensory indicator for consumers to evaluate the quality of THHJ.

#### 3.3.3. Odor Activity

**Figure 5:**
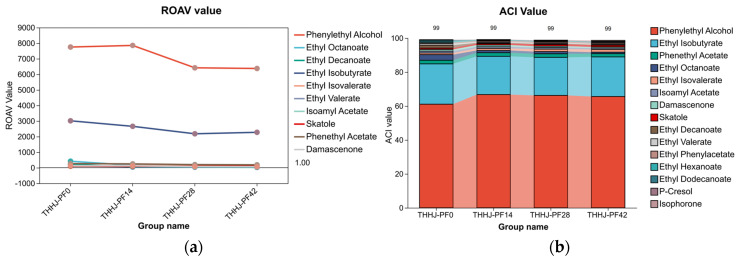
ROAV (air) of VFCs in THHJ during the post-fermentation process (**a**); ACI value of VFCs (**b**).

One of the approaches for evaluating odor intensity involves the threshold test. The threshold value is defined as the minimum perceptible odor concentration, which is determined via the dilution of a specified amount of sample using odorless and tasteless compounds (e.g., air or water) as solvents, and its reciprocal is utilized to represent odor intensity. The relative odor activity value (ROAV) enables the quantitative assessment of the contribution of different volatile compounds to the overall flavor, thereby facilitating the identification of key flavor-active compounds. To date, numerous studies have reported the application of this method in the investigation of Chinese rice wine [39]. Furthermore, the Aroma Character Impact Value (ACI) serves as an indicator employed in food flavor research to quantify the contribution of individual volatile components to the overall aroma profile [40], and it is frequently used in combination with ROAV for comprehensive analytical purposes. In the present study, ROAV (with air as the medium) was employed to evaluate the contribution of specific compounds to the aroma characteristics of THHJ during the post-fermentation process. Odor threshold values were retrieved from published literature data [41,42]. Characteristic aroma compounds in THHJ post-fermentation samples were screened based on the criterion of ROAV > 1. In addition, ACI was calculated using the formula (1):

(1) $$\mathrm{ACI}\%=\frac{{OT}_{x}}{{OT}_{t}}\times 100$$

where *OT_x_* is the odor threshold of the *x*th compound, and *OT_t_* is the sum of odor thresholds of all compounds, in which a higher ACI value denotes a greater contribution of the corresponding compound to the overall aroma. As illustrated in Figure 5, the ROAV of phenylethyl alcohol (range: 6376–7856) was markedly higher than that of other compounds, followed by ethyl isobutyrate (range: 2277–3051). This demonstrates that the most intense odor emitted by THHJ is primarily derived from phenylethyl alcohol and ethyl isobutyrate. Consistent with the ROAV results, the ACI values of phenylethyl alcohol (range: 61.10–66.74%) and ethyl isobutyrate (range: 22.45–23.74%) were considerably higher than those of other compounds. These findings further verify that the contents of these two compounds largely dictate the overall sensory properties of THHJ, which may provide a potential indicator for producers in quality monitoring.

#### 3.3.4. Association Between Key Flavor Metabolites and Microbial Community

**Figure 6:**
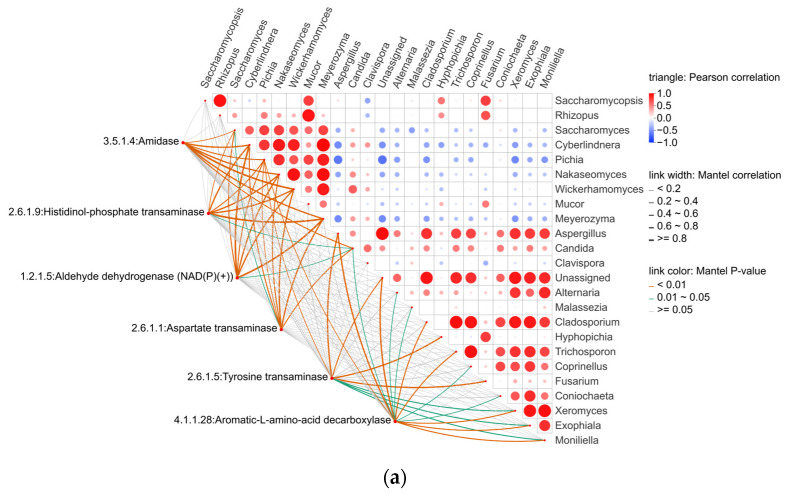

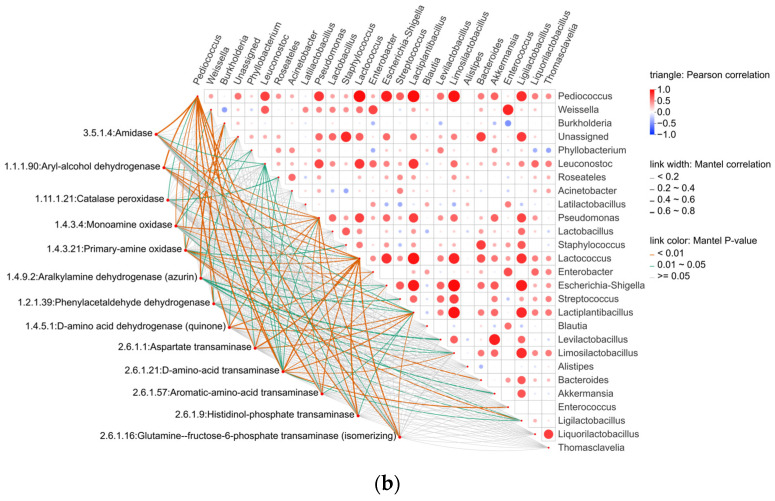
Correlation analysis between microorganisms and phenylethyl alcohol biosynthetic enzymes: Mantel test heatmap of fungal absolute abundance values with predicted enzyme abundance (**a**); Mantel test heatmap of bacterial absolute abundance values with predicted enzyme abundance (**b**). The orange line represents a statistically significant positive correlation, and the green line represents a negative correlation.

It is well established that the flavor of alcoholic beverages is closely associated with the microorganisms involved in fermentation [43]. Previous studies have reported correlation analyses between the abundance of flavor compounds and microbial communities within the same samples during the fermentation of Chinese rice wine [27,44]. However, in the brewing process of THHJ, changes in microbial community structure are concentrated in the saccharification stage, whereas alterations in volatile flavor compounds (VFCs) primarily occur during post-fermentation. This temporal asynchrony precludes direct correlation analysis between microbial communities and VFCs. Nevertheless, VFCs are recognized as microbial metabolites, and previous research has identified key VFCs in THHJ, including phenylethyl alcohol, ethyl acetate, and ethyl lactate. Notably, the enzymes involved in the metabolic pathways of these metabolites (or their precursors) remain functionally active even when the corresponding microorganisms are inhibited. In light of this, the present study employed PICRUSt (Phylogenetic Investigation of Communities by Reconstruction of Unobserved States) to predict the metabolic functions of microbial communities during saccharification. Specifically, 16S rRNA and ITS2 gene sequencing data were compared against reference genome databases of microorganisms with well-characterized metabolic functions, enabling the prediction of bacterial and fungal metabolic capabilities. Subsequently, enzymes involved in phenylethyl alcohol synthesis within the KEGG (Kyoto Encyclopedia of Genes and Genomes) phenylalanine metabolic pathway (KEGG map: 00360) were screened. Correlation analyses between the absolute abundances of fungi/bacteria and the predicted abundances of these enzymes were performed using the Two-way Orthogonal Partial Least Squares (O2PLS) model combined with Pearson correlation coefficients, integrated with KEGG pathway data. The predicted abundances of enzymes associated with phenylethyl alcohol synthesis in the phenylalanine metabolic pathway are presented in Table 1 and Table 2. A total of 6 fungal-associated enzymes and 13 bacterial-associated enzymes were screened, among which the predicted abundance of Aryl-alcohol dehydrogenase (EC 1.1.1.90), directly involved in phenylethyl alcohol biosynthesis, was significantly higher than that of other enzymes and was attributed to bacterial communities. The results of correlation analyses between the predicted enzyme abundances and microbial absolute abundances are illustrated in Figure 6. Specifically, enzymes involved in the upstream of the phenylethyl alcohol metabolic pathway, such as Aldehyde dehydrogenase (NAD(P)(+)) (EC 1.2.1.5), exhibited significantly positive correlations with *Saccharomyces*, *Cyberlindnera*, *Pichia*, *Nakaseomyces*, *Wickerhamomyces* and *Meyerozyma* (Figure 6a). Meanwhile, enzymes such as Aryl-alcohol dehydrogenase (EC 1.1.1.90) showed significantly positive correlations with *Pediococcus*, *Pseudomonas*, *Lactococcus,* and *Lactiplantibacillus* (Figure 6b). Previous studies have shown that the biosynthesis of phenylethyl alcohol in Huangjiu is closely related to *Saccharomyces* and non-*Saccharomyces* yeasts in “Jiuqu” [45]. Other studies have also indicated that the biosynthesis of phenylethyl alcohol is associated with a variety of bacteria [46]. The findings of this study suggest that phenylethyl alcohol in THHJ is a product of the combined action of multiple microorganisms, rather than a simple association between metabolite concentrations and microbial relative abundances. This study facilitates the identification of links between volatile flavor compounds and microbial communities in THHJ, providing insights into the underlying flavor formation mechanisms.

### 3.4. Metabolomics Changes in THHJ During Post-Fermentation Process

#### 3.4.1. Metabolite Analysis

**Figure 7:**
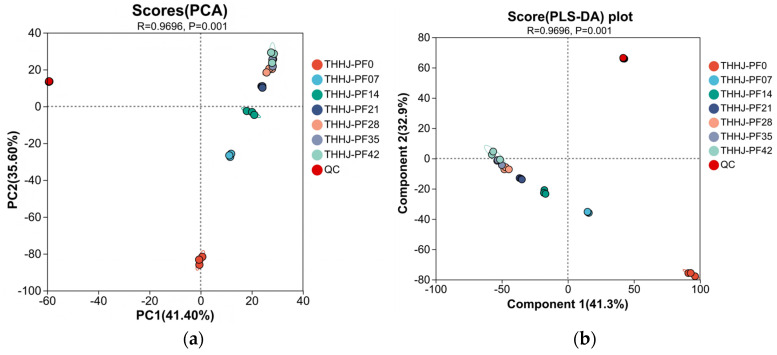

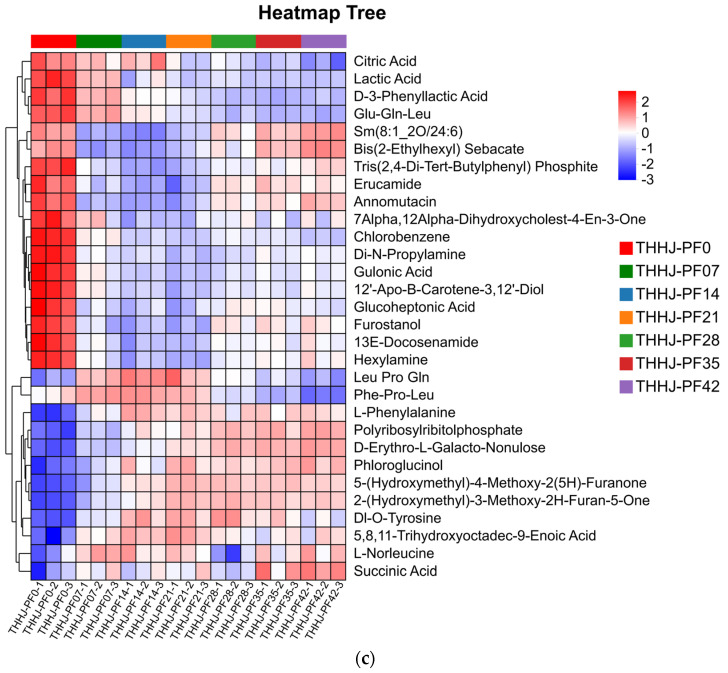

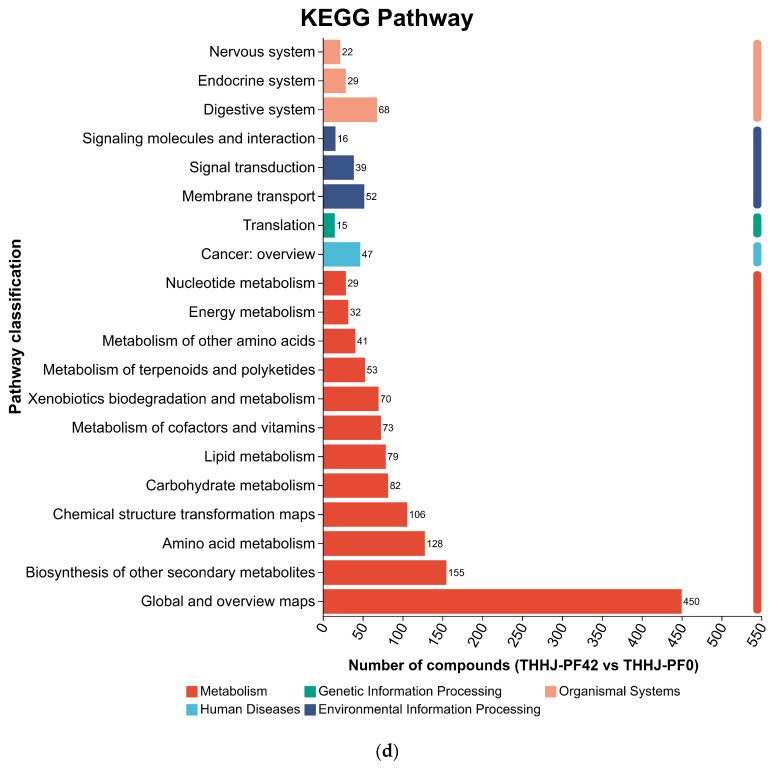
PCA score plot (**a**); PLS-DA score plot (**b**); Heatmap of metabolite-sample relationships (**c**); Statistical analysis of different metabolite counts in KEGG pathways (THHY-PF42_vs_THHY-PF0) (**d**).

In the present study, a total of 4438 metabolites were detected and identified during the post-fermentation process (Spreadsheet S2). Principal Component Analysis (PCA) score plot (Figure 7a) indicated that Principal Component 1 (PC1) and Principal Component 2 (PC2) explained 41.40% and 35.60% of the total variance, respectively. Samples at 7 days (THHJ_PF07) and 14 days (THHJ_PF14) of post-fermentation were distinctly separated from the initial sample (THHJ_PF0), whereas samples fermented for 28 (THHJ_PF28), 35 (THHJ_PF35), and 42 days (THHJ_PF42) clustered closely with minimal inter-group variation. These observations confirmed that the metabolome of THHJ underwent substantial changes within the first 7 days of post-fermentation, with the rate of metabolomic alteration decreasing between 7 and 28 days and metabolites stabilizing after 28 days. Given that the typical post-fermentation period for THHJ is ~30 days in industrial production, an extended fermentation beyond this duration is deemed unnecessary, and the PCA results are consistent with practical manufacturing requirements. Figure S2 summarizes the number of significantly differential metabolites (SDMs) between each post-fermentation time point and the initial stage (THHJ_PF0). Even though most metabolites exhibited no significant changes, the continuous presence of SDMs indicated that the metabolic system remained dynamically active during post-fermentation. Hierarchical clustering analysis (HCA) of the top 50 SDMs (Figure 7c) revealed the accumulation of organic acids, amino acids, dipeptides, tripeptides, alkaloids, and biogenic amines during post-fermentation. Since peptides and amino acids are primarily derived from the protease-mediated hydrolysis of macromolecular proteins, these results suggest that microbial enzymes retain catalytic activity despite the high alcohol concentration in the post-fermentation matrix, which inhibits microbial viability. Volcano plots of SDMs from multi-group comparisons (Figure S2, highlighting the top 5 metabolites by FC value) identified key compounds: Conferone (*p* = 2.977 × 10^−11^, most significant difference), Neoechinulin A (FC = 97.0867, highest upregulation), Pc (Pgf1Alpha/2:0) (presumably PC (2:0/PGF1alpha), an oxidized phosphatidylcholine, FC = 0.1106, highest downregulation), and Icariside D1 (consistently increasing FC, indicating a gradual rise in relative content during fermentation). Potential misidentification of some compounds cannot be ruled out due to the structural similarity of organic molecules and the technical limitations of the detection platform, warranting further validation and characterization in future studies. Additionally, the number of compounds annotated by the KEGG data base and the distribution of SDMs across KEGG pathways are presented in Figure 7d “Metabolic pathways” contained the largest number of annotated compounds, with “Global and overview maps” accounting for the highest proportion, followed by pathways involved in the biosynthesis and metabolism of secondary metabolites, cofactors, vitamins, and amino acids. These metabolites are predominantly plant-specific secondary metabolites or minor fermentation-derived compounds rather than core metabolic intermediates, suggesting their potential as novel functional markers for THHJ metabolomics research. In future work, to elucidate the correlations between metabolites, microbial communities, and enzymomes, and will focus on characterizing the key microorganisms and functional enzymes involved in flavor formation and bioactive compound production in THHJ. This will include investigating their metabolic mechanisms and critical pathways, with the aim of identifying functional production strains and probiotics for industrial application.

#### 3.4.2. Changes in Organic Acids During Post-Fermentation Process

**Figure 8:**
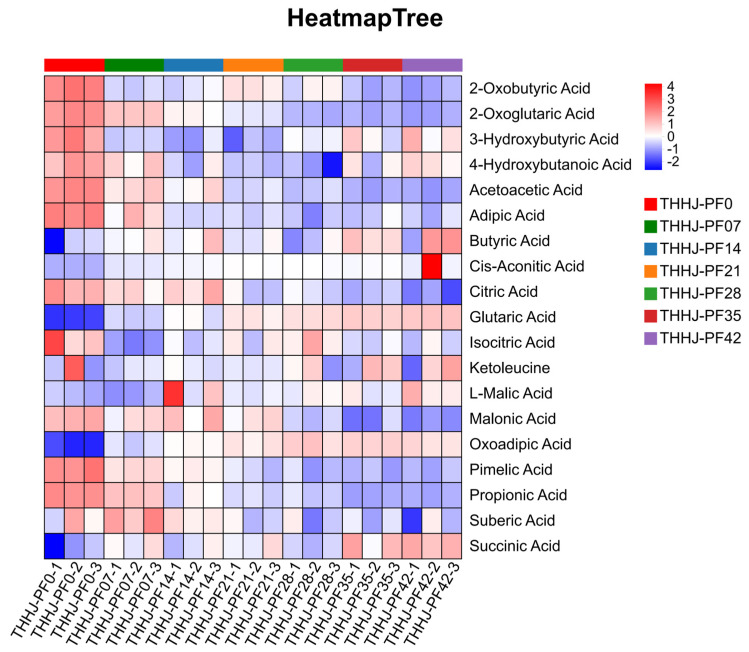
Hierarchical clustering analysis for the significantly differentially regulated organic acids of THHJ during post-fermentation.

Changes in various organic acids during the post-fermentation process of THHJ were systematically quantified via LC-MS, and the identified metabolites were annotated using the KEGG database. These metabolites were further filtered through *p*-value and OPLS-DA analysis. Only compounds satisfying the criteria of *p* < 0.05 and VIP > 1 displayed significant variations in relative content after 42 days of fermentation. As shown in Figure 8, the level of glutaric acid in THHJ was notably elevated following the 42-day post-fermentation period. For other KEGG-classified organic acids, some exhibited *p*-values far below 0.05; however, their VIP values from the OPLS-DA analysis were below 1, indicating a relatively limited impact of their changes on the overall post-fermentation process, which was not reported by any previous research. Additionally, the present study monitored dynamic changes in approximately 500 acidic compounds (Figure S3a) during post-fermentation. These compounds, though not categorized as organic acids in KEGG, possess acid moieties, and their structural compositions are presented in Figure S3b. Importantly, these acid moiety-containing compounds represent the major contributors to total acidity in THHJ. Typically, acidic compounds not only impart a sour taste but also function as precursors for ester synthesis. During THHJ aging, they react with alcohols to form esters; however, esterification proceeds slowly under the mild aging conditions (i.e., no heating or artificial catalysts added), which accounts for the prolonged period required for natural aging to yield high-quality Huangjiu [47]. In contrast, THHJ undergoes a relatively short post-fermentation period of only 1–2 months, resulting in minimal consumption of acidic compounds and thus no significant difference in their contents before and after post-fermentation. Furthermore, neither total acid content nor pH exhibited significant variations during the post-fermentation stage (Figure 1b), which aligns with the omics analysis results of acidic compounds. This suggests that the majority of acidic compounds are generated during the saccharification stage by various acid-producing microorganisms. During post-fermentation, microbial activity diminishes and eventually ceases. Consequently, the content of most microbially synthesized acidic compounds in THHJ stops increasing, begins to convert into esters or other derivatives, and gradually decreases. Although various organic acids (e.g., citric acid and malic acid) act as common flavor contributors in THHJ, their more critical role is to promote ester formation and facilitate the development of THHJ’s unique flavor profile. For instance, lactic acid and acetic acid, two of the most vital organic acids in THHJ, serve as precursors for ethyl lactate and ethyl acetate, which are key flavor components of THHJ (Figure 4c). Moreover, several of these acids also possess bioactive properties. For instance, ferulic acid [48] is widely acknowledged as a health-promoting compound due to its potent antioxidant activity [49], cardioprotective effects, anti-inflammatory properties, and immunomodulatory capabilities, whereas ganosporeric acid A, ganoderic acid K, and lucidenic acid E2 demonstrate potential anti-inflammatory and antiviral activities [50,51,52].

#### 3.4.3. Changes in Amino Acid and Peptide During Post-Fermentation Process

**Figure 9:**
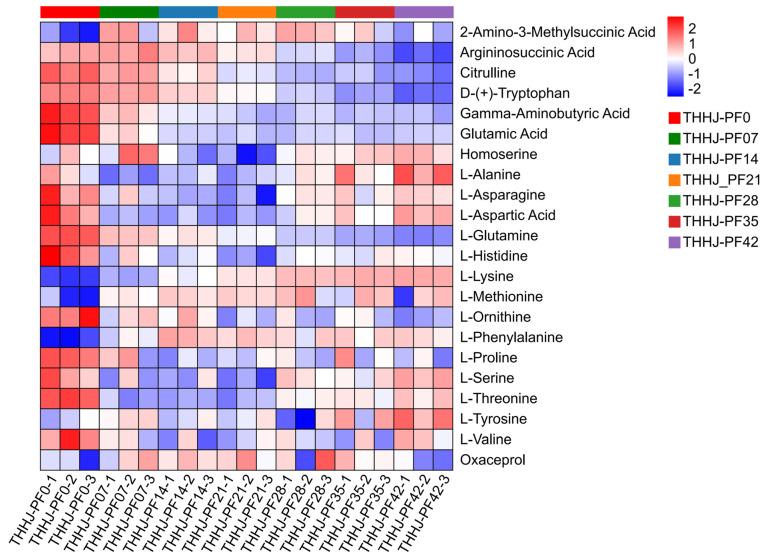
Hierarchical clustering analysis for the significantly differentially regulated amino acids of THHJ during post-fermentation.

Peptides, amino acids, and their derivatives represent the primary sources of amino acid nitrogen (AAN) in fermented commodities, including soy sauce, Chinese rice wine, and aged vinegar [53]. As a critical quality indicator, AAN content is widely employed to assess the fermentation maturity of such products, with higher AAN levels generally correlating with superior product quality. In THHJ, the overwhelming majority of peptides and amino acids are derived from the hydrolysis of glutinous rice-derived proteins through the action of microbial proteases. Notably, a subset of these amino acids belongs to the non-protein amino acid category, which may originate either from the glutinous rice raw material or metabolic pathways of the fermenting microflora [54]. The gustatory properties of amino acids are predominantly governed by the structural characteristics of their side chains (R-groups), while being modulated by additional factors such as stereoconfiguration, pH value, concentration, and intermolecular interactions [55,56,57]. The majority of free amino acids identified in this study exhibited the L-configuration, and their temporal dynamics are detailed in Figure 9. After 42 days of fermentation, most free amino acids displayed non-significant variations (*p* < 0.05, VIP < 1), with only L-glutamine, citrulline, and D-(+)-tryptophan undergoing significant downregulation. This non-significant fluctuation pattern suggests that these free amino acids are primarily biosynthesized during the saccharification process, mediated by the hydrolytic activity of microbial proteases. Prior studies have delineated the specific taste contributions of individual amino acids in rice wine [58,59]. However, the proteolytic cascade from proteins to free amino acids generates a diverse array of intermediate peptides, and these amino acid-residue assemblies may exert profound impacts on THHJ flavor. Consequently, this study systematically characterized peptide profiles during post-fermentation, with their dynamic changes illustrated in Figure S3a. A total of 1124 peptides with differential abundance were detected: at 7 days of post-fermentation, 974 peptides exhibited upregulated relative abundance, whereas 147 peptides showed downregulation. As post-fermentation progressed, the number of upregulated peptides declined concomitantly with an increase in downregulated counterparts. This phenomenon is likely attributed to the gradual attenuation of protease hydrolytic activity over extended post-fermentation periods. The length distribution of the detected peptides is presented in Figure S3b, with dipeptides, tripeptides, tetrapeptides, and pentapeptides accounting for 16.21%, 75.87%, 4.81%, and 2.23%, respectively. Additionally, 10 peptides containing six or more amino acid residues were identified. As shown in Figure S3c, the amino acid composition of these peptides was dominated by hydrophobic residues: Leu (9.9%), Phe (8.67%), Ile (8.08%), and Val (7.7%), collectively contributing 36.37%. These hydrophobic amino acids constitute the primary components of short-chain peptides (particularly tripeptides and dipeptides) and are thus hypothesized to be directly associated with the characteristic flavor profiles of rice wine, including mellowness and bitterness. Furthermore, Glu (8.01%), a canonical umami amino acid, likely constitutes one of the key contributors to the umami taste of rice wine due to its high prevalence in peptides. Aromatic amino acids, including Phe, Tyr (4.39%), and Trp (1.69%), accounted for 15.63% of the total residues. The aromatic ring structures of these amino acids may serve as precursors for unique aromatic compounds [60] in rice wine or undergo oxidation and degradation to generate flavor-active metabolites, thereby enhancing flavor complexity. Notably, while the taste of individual amino acids is primarily dictated by their side-chain structures, the gustatory activity of peptides is not a mere additive effect of constituent amino acid side chains [61]. Instead, it is synergistically determined by side-chain combinations, peptide chain length, and spatial conformation. Short-chain peptides (dipeptides and tripeptides) exhibit robust gustatory activity owing to their fully exposed side chains, whereas long-chain peptides display diminished taste activity as their side chains are sterically shielded by peptide bonds. Sensory flavor profile analysis revealed that THHJ predominantly exhibits rose, sweet, bitter, fruity, green, and balsamic notes (Figure 4a), which are primarily attributed to sugars, esters, phenylethanol, and fusel oils. Studies on the flavor contributed by peptides have mostly focused on sauce condiments and fermented soybean products, particularly on salty peptides [62] and umami peptides [63], whereas relevant reports remain scarce in Huangjiu. Given the discrepancy between peptide/amino acid distribution and sensory characteristics, the transformation kinetics of peptides and amino acids during THHJ post-fermentation, as well as the magnitude of their contributions to THHJ taste, warrant further systematic investigation.

#### 3.4.4. Changes in Functional Compounds During Post-Fermentation Process

**Figure 10:**
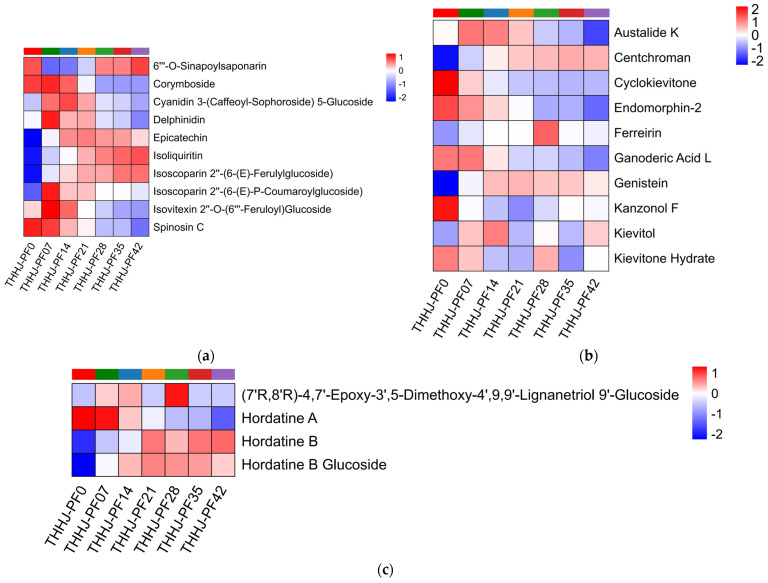

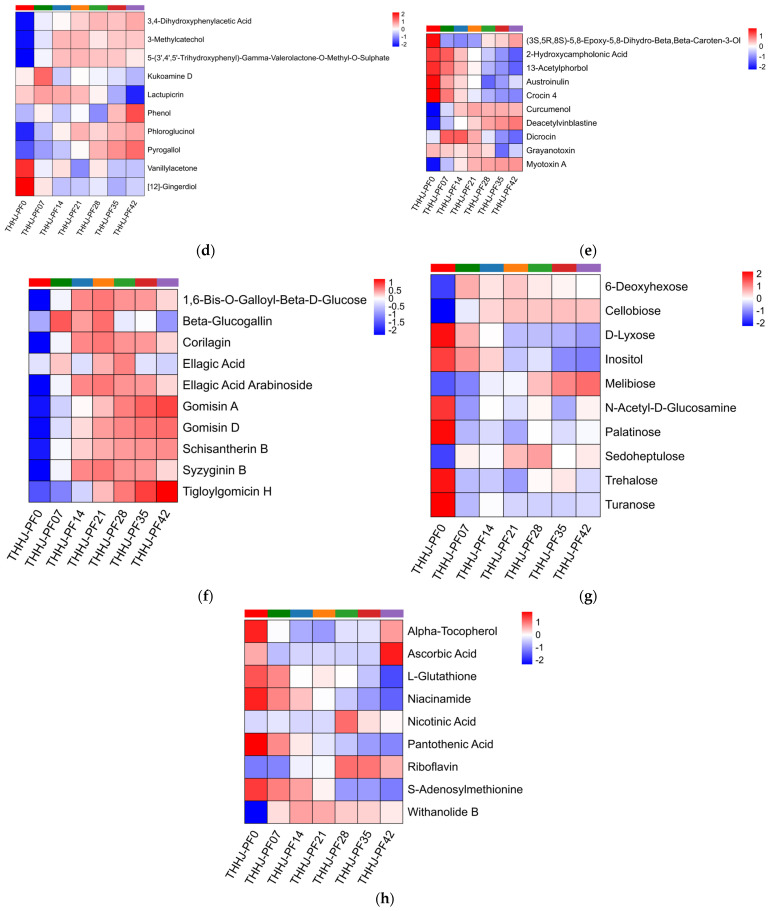

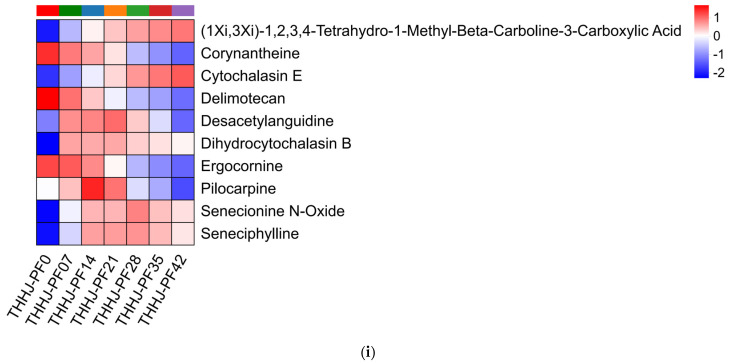
Hierarchical clustering analysis for the significantly differentially regulated functional compounds of THHJ during post-fermentation. Flavonoids (**a**), isoflavonoids (**b**), 2-arylbenzofuran flavonoids (**c**), phenols (**d**), terpenoids (**e**), tannins (**f**), saccharides (**g**), vitamins (**h**), alkaloids (**i**).

Previous studies have demonstrated that Chinese rice wine contains a variety of bioactive functional compounds, which confer potential health-beneficial properties to it, such as anti-inflammatory, antioxidant, antiviral, antitumor activities, cardiovascular and cerebrovascular health maintenance, and immune regulation. As a subtype of Chinese rice wine, THHJ also exhibits certain health-promoting functionalities [10]. Drawing on previous research on functional compounds in THHJ and other Chinese rice wines, the present study screened and categorized 9 classes of compounds from the metabolomic detection results. These compounds were annotated using the KEGG and HMDB databases, including 75 flavonoids, 10 isoflavonoids, 4 2-arylbenzofuran flavonoids, 21 phenols, 171 terpenoids, 11 tannins, 17 saccharides, 6 vitamins and cofactors, and 21 alkaloids and their derivatives. The specific variations in each class of compounds after 42 days of post-fermentation are presented in Figure 10. Among these compounds, S-adenosylmethionine (a cofactor) exhibited the highest VIP value (VIP = 2.67, *p* = 3.45 × 10^−9^, significantly upregulated), while alpha-bixin (a diterpenoid) showed the most significant difference (*p* = 1.48 × 10^−9^, minimum *p*-value). 25-Acetylvulgaroside (a sesterterpenoid) had the maximum upregulation fold change (FC = 2.28), and lactupicrin (a phenol) displayed the maximum downregulation fold change (FC = 0.42). Flavonoids and terpenoids are the major functional compounds in THHJ. As shown in Figure 8, the FC values of most flavonoids were more concentrated around 0 compared with those of terpenoids, indicating that the variations in flavonoids during the post-fermentation of THHJ were less pronounced than those of terpenoids. It is speculated that most flavonoids in THHJ may be derived from raw materials and have a weaker association with microbial metabolic processes than terpenoids. Nevertheless, microorganisms can indeed induce the transformation of flavonoids in food raw materials and generate other functional compounds [64]. Terpenoids are a class of compounds with both specific flavor and functional properties. To date, studies on terpenoids have been conducted in grape wine, other fruit wines [65], and Chinese Baijiu [66], whereas research related to terpenoids in Chinese rice wine remains limited. Existing studies have confirmed that microorganisms affect the content of terpenoids in alcoholic beverages [67], and the optimization of fermentation strains can increase the abundance of terpenoids [68], thereby improving the flavor of the wine. This evidence confirms that terpenoids in wine are indeed associated with microbial metabolism. Accordingly, the correlations between these functional compounds and microorganisms in THHJ are worthy of further investigation.

#### 3.4.5. Correlations Between Functional Compounds and Microbial Communities

**Figure 11:**
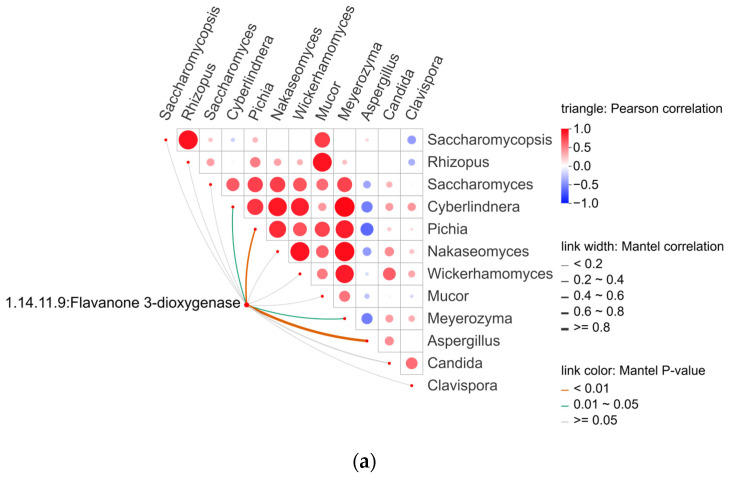

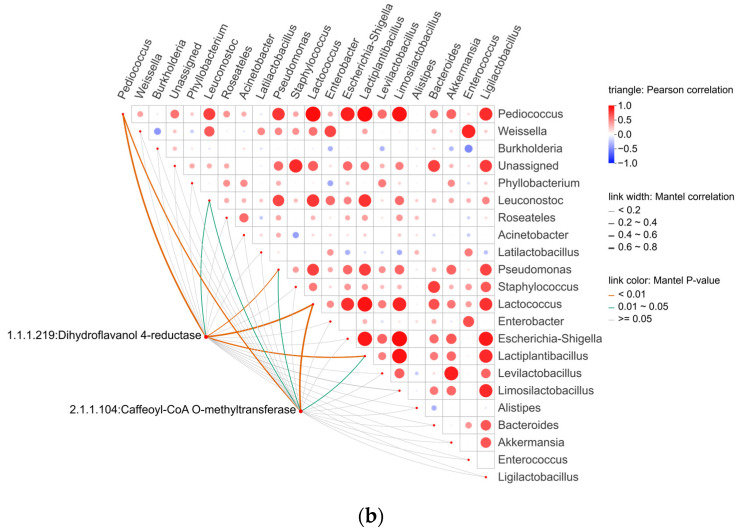
Correlation analysis between microorganisms and flavonoid biosynthetic enzymes: Mantel test heatmap of fungal absolute abundance values with predicted enzyme abundance (**a**); Mantel test heatmap of bacterial absolute abundance values with predicted enzyme abundance (**b**). The orange line represents a statistically significant positive correlation; the green line represents a negative correlation.

Although the functional compounds of Chinese rice wine have been extensively investigated, research on their associations with microorganisms remains limited. In contrast to VFCs, these functional compounds may originate from raw materials [69]. On the other hand, similar to VFCs, functional compounds produced via microbial metabolism in THHJ cannot be directly linked to specific microorganisms. Furthermore, owing to the high diversity of functional compounds in THHJ—with multiple structurally distinct compounds within the same metabolite class—and the complexity of their metabolic networks, the number of enzymes involved in a single metabolic pathway can be substantial. This includes not only enzymes directly responsible for synthesizing the target compound but also those participating in upstream and downstream metabolic pathways. To date, no robust methodology has been established for the unified analysis of these compounds; consequently, the present study focused exclusively on examining the correlation between the flavonoid biosynthesis pathway and microorganisms. Table 3 presents the predicted abundances (determined via PICRUSt2) of four key enzymes involved in three pathway modules (M00137, M00138, M00940) of the flavonoid biosynthesis pathway (ko00941) during the saccharification stage of THHJ production. Correlation analysis between the predicted enzyme abundances and microbial abundances is depicted in Figure 11, which demonstrates that one enzyme exhibited significantly positive correlations with 2 fungal genera (*Aspergillus*, *Pichia*), while the other two enzymes showed significantly positive correlations with 4 bacterial genera. Integrated analysis of the predicted enzyme abundances and correlation data indicates that the biosynthesis of specific flavonoids in THHJ may be closely associated with bacterial genera, including *Pediococcus*, *Pseudomonas*, *Lactococcus*, and *Lactiplantibacillus*. Notably, several species within these bacterial genera have been reported to exhibit probiotic properties [70,71,72], and their utilization in the fabrication of functional fermented foods has been investigated [73,74,75]. The production of functional metabolites during the transformation of raw food materials is one of the core mechanisms underlying the probiotic effects of these microorganisms. However, different raw material varieties (rice) [76] contain distinct natural constituents. These constituents act as substrates for microorganisms and are converted into different end products, resulting in variations in the types and contents of functional components present in the final Huangjiu products. Similarly, these probiotic microorganisms may contribute to the functional properties of THHJ during its production process, ultimately endowing THHJ with its unique functional characteristics. Future research will focus on other types of functional compounds in THHJ and their correlations with microorganisms. This work aims to provide theoretical insights for the screening of probiotic strains from the THHJ production process and the optimization of the functional characteristics of THHJ as a functional food.

## 4. Conclusions

This study preliminarily revealed the dynamic changes in fermentative microbiology and microbial metabolites of THHJ during its production process. Moreover, the correlations between the metabolic pathways of volatile metabolites, functional compounds, and the corresponding microbial communities were explored. Microbiome analysis demonstrated that the dominant microbial genera in THHJ during saccharification were *Pediococcus*, Saccharomycopsis, *Rhizopus*, *Weissella*, and *Limosilactobacillus*. Volatile flavor metabolomics profiling of THHJ during post-fermentation identified phenylethyl alcohol, ethyl acetate, ethyl lactate, and isoamyl acetate as the key flavor-active compounds. Notably, phenylethyl alcohol was the most pivotal, as its content in THHJ dictated the overall sensory properties, and its biosynthesis was closely associated with a variety of bacterial and fungal taxa. Metabolomics analysis revealed dynamic alterations in a diverse array of complex biological metabolites during THHJ post-fermentation. Organic acids and acid radical-bearing compounds were detected, alongside a range of peptides beyond amino acids. Furthermore, substantial changes in multiple functional compounds were noted during post-fermentation, among which the metabolism of flavonoids was correlated with microbes exhibiting potential probiotic activity, such as *Pediococcus*, *Lactococcus*, and *Lactiplantibacillus*. These findings offer reference data and technical insights for the development and optimization of THHJ fermentation technologies, as well as the exploration of probiotic resources.

## Figures and Tables

**Table 1:** Predicted enzyme abundance in fungi.

EC_Function	THHJ_S0	THHJ_S24	THHJ_S48	THHJ_S72	THHJ_S96
1.2.1.5:Aldehyde dehydrogenase (NAD(P)(+))	5133	5144 ^ns^	11,462 *	7350 ^ns^	16,886 ***
2.6.1.1:Aspartate transaminase	8331	5209 ^ns^	11,800 ^ns^	7481 ^ns^	17,038 **
2.6.1.5:Tyrosine transaminase	1067	23 ****	60 ****	42 ****	69 ****
2.6.1.9:Histidinol-phosphate transaminase	2583	2578 ^ns^	5837 *	3690 ^ns^	8441 ***
3.5.1.4:Amidase	14,729	10,428 ^ns^	23,348 ^ns^	14,980 ^ns^	34,110 **
4.1.1.28:Aromatic-L-amino-acid decarboxylase	1530	63 ***	235 **	78 ***	115 **

ns: *p* > 0.05; *: *p* ≤ 0.05; **: *p* ≤ 0.01; ***: *p* ≤ 0.001; ****: *p* ≤ 0.0001 (THHJ_S0 group compare with other groups).

**Table 2:** Predicted enzyme abundance in bacteria.

EC_Function	THHJ_S0	THHJ_S24	THHJ_S48	THHJ_S72	THHJ_S96
1.1.1.90:Aryl-alcohol dehydrogenase	1.70 × 10^5^	3.97 × 10^7^ **	1.27 × 10^8^ ****	5.40 × 10^7^ ***	8.02 × 10^7^ ****
1.11.1.21:Catalase peroxidase	10,330	38,844 ^ns^	38,038 ^ns^	16,140 ^ns^	29,738 ^ns^
1.2.1.39:Phenylacetaldehyde dehydrogenase	26,941	30,860 ^ns^	40,247 ^ns^	14,649 ^ns^	24,128 ^ns^
1.4.3.21:Primary-amine oxidase	197	602 ^ns^	1263 ^ns^	2173 *	2693 **
1.4.3.4:Monoamine oxidase	2076	8938 ^ns^	17,455 ^ns^	5071 ^ns^	12,315 ^ns^
1.4.5.1:D-amino acid dehydrogenase (quinone)	2.19 × 10^5^	3.97 × 10^7^ **	1.26 × 10^8^ ****	5.39 × 10^7^ ***	8.00 × 10^7^ ****
1.4.9.2:Aralkylamine dehydrogenase (azurin)	1.74 × 10^4^	16,215 ^ns^	15,367 ^ns^	4415 ^ns^	8063 ^ns^
2.6.1.1:Aspartate transaminase	1.74 × 10^5^	3.67 × 10^7^ **	1.20 × 10^8^ ****	5.02 × 10^7^ ***	7.61 × 10^7^ ****
2.6.1.16:Glutamine--fructose-6-phosphate transaminase (isomerizing)	2.72 × 10^5^	4.02 × 10^7^ **	1.29 × 10^8^ ****	5.87 × 10^7^ ***	8.63 × 10^7^ ****
2.6.1.21:D-amino-acid transaminase	1.63 × 10^4^	9.25 × 10^4 ns^	6.65 × 10^5 ns^	4.08 × 10^6^ ****	4.98 × 10^6^ ****
2.6.1.57:Aromatic-amino-acid transaminase	8.97 × 10^4^	3.63 × 10^6^ **	9.98 × 10^6^ ****	4.76 × 10^6^ ***	5.72 × 10^6^ ****
2.6.1.9:Histidinol-phosphate transaminase	3.01 × 10^4^	1.06 × 10^5^ *	2.01 × 10^5^ ***	1.41 × 10^5^ **	1.85 × 10^5^ ***
3.5.1.4:Amidase	1.20 × 10^5^	3.75 × 10^6^ **	1.04 × 10^7^ ****	5.07 × 10^6^ ***	6.05 × 10^6^ ****

ns: *p* > 0.05; *: *p* ≤ 0.05; **: *p* ≤ 0.01; ***: *p* ≤ 0.001; ****: *p* ≤ 0.0001 (THHJ_S0 group compare with other groups).

**Table 3:** Predicted enzyme abundance in fungi and bacteria.

EC_Function	THHJ_S0	THHJ_S24	THHJ_S48	THHJ_S72	THHJ_S96
Fungi					
1.14.11.9:Flavanone 3-dioxygenase	2150	53 ****	129 ****	64 ****	87 ****
					
Bacteria					
1.1.1.219:Dihydroflavanol 4-reductase	18,469	131,576 ^ns^	518,786 ****	282,359 ***	379,815 ****
2.1.1.104:Caffeoyl-CoA O-methyltransferase	1125	97,793 ^ns^	478,729 ****	329,956 ****	368,950 ****

ns: *p* > 0.05; ***: *p* ≤ 0.001; ****: *p* ≤ 0.0001 (THHJ_S0 group compare with other groups).

## Data Availability

The original contributions presented in this study are included in the article/Supplementary Material. Further inquiries can be directed to the corresponding author.

## Supplementary Materials

The following supporting information can be downloaded at: https://www.mdpi.com/article/10.3390/foods15060999/s1, Figure S1. Scatter plot of differential volatile compounds during post-fermentation process of THHJ: Experimental group (EG) vs. control Group (CG); Figure S2. Scatter plot of differential metabolites during the post-fermentation process of THHJ: Experimental group (EG) vs. control Group (CG); Figure S3. Scatter plot of compounds bearing acid radicals (HMDB annotation) during the post-fermentation process (a); Classification of compounds bearing acid radicals (HMDB super class) (b); Figure S4. Scatter plot of peptide during post-fermentation process (a); Types of peptides (b); Amino acid residues in peptides (c); Spreadsheet S1: Information of volatile organic compounds; Spreadsheet S2: Information of metabolites. The Supplementary Materials can also be downloaded at: https://doi.org/10.5281/zenodo.18333255 (accessed on 20 January 2026)
