# Supplementary figures and images for: Microbial Communities, Volatile Flavor Profiles and Metabolomic Characteristics During Traditional Hakka Huangjiu Fermentation
Source: Foods. 2026 Mar 11;15(6):999. doi: 10.3390/foods15060999 (PMC13025649; doi:10.3390/foods15060999)

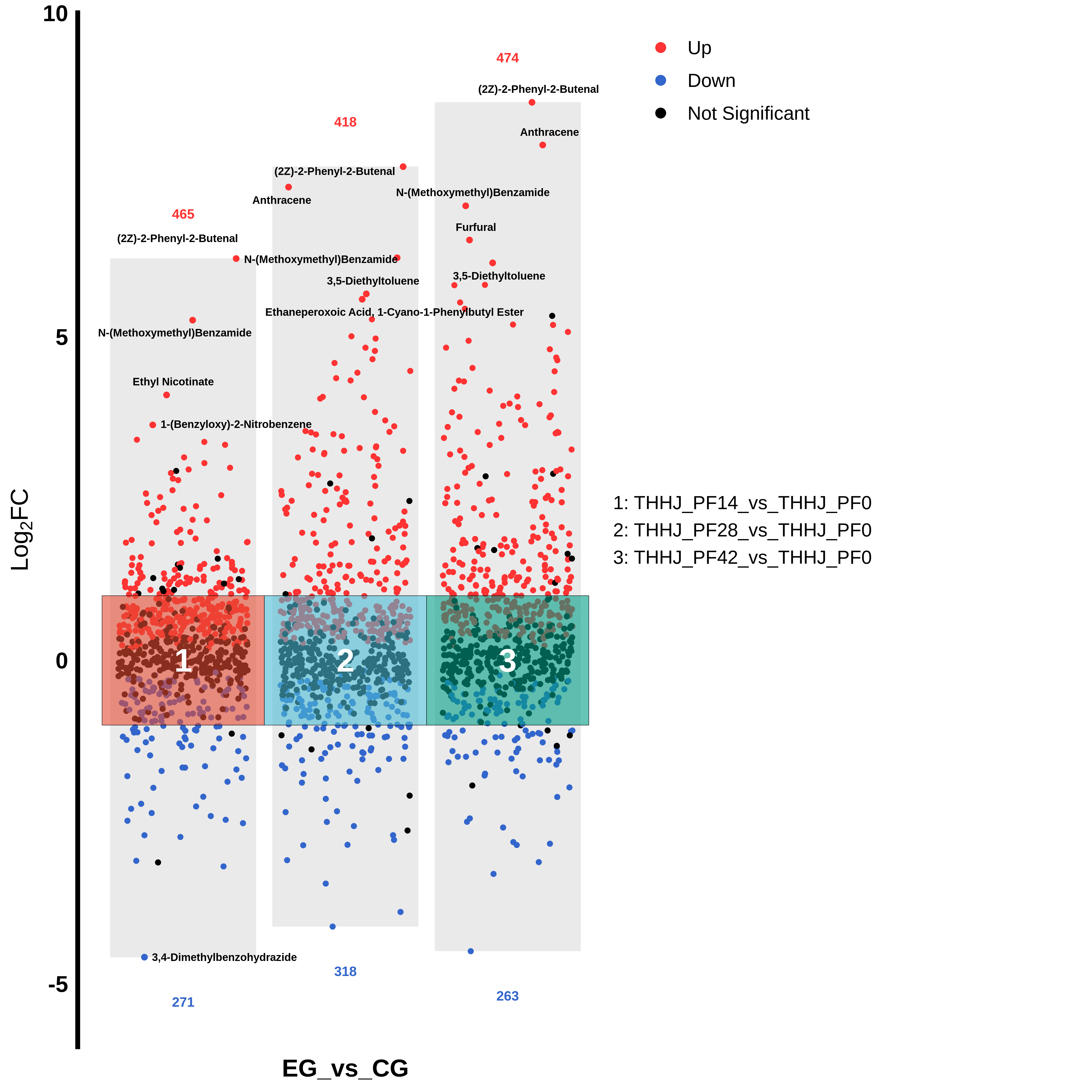

Supplement: Supplementary file 1 [file foods-15-00999-s001.zip › Figure S1_Scatter plot of differential volatile compounds during post-fermentation process of THHJ.png]

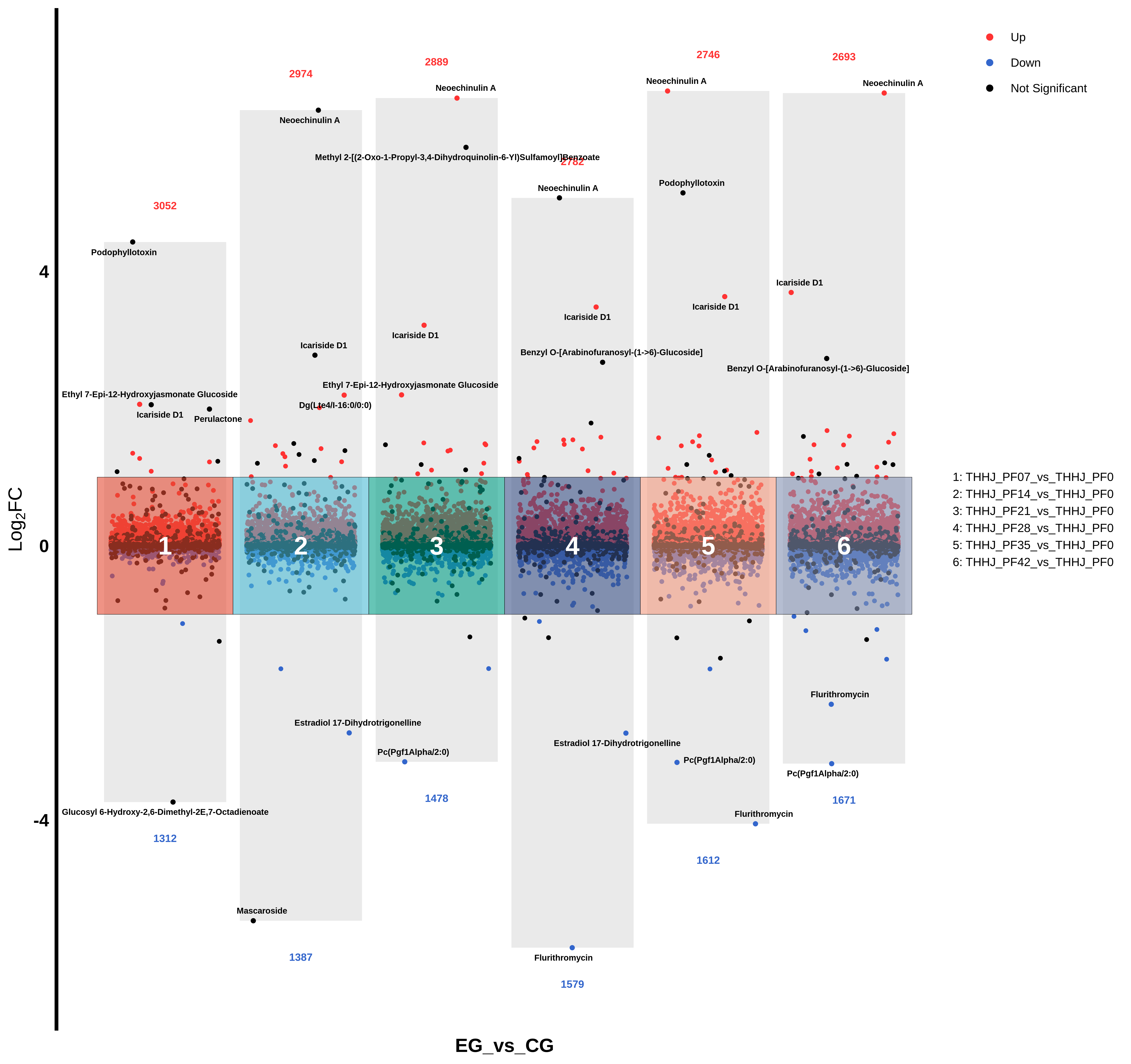

Supplement: Supplementary file 1 [file foods-15-00999-s001.zip › Figure S2_Scatter plot of differential metabolites during post-fermentation process of THHJ.png]

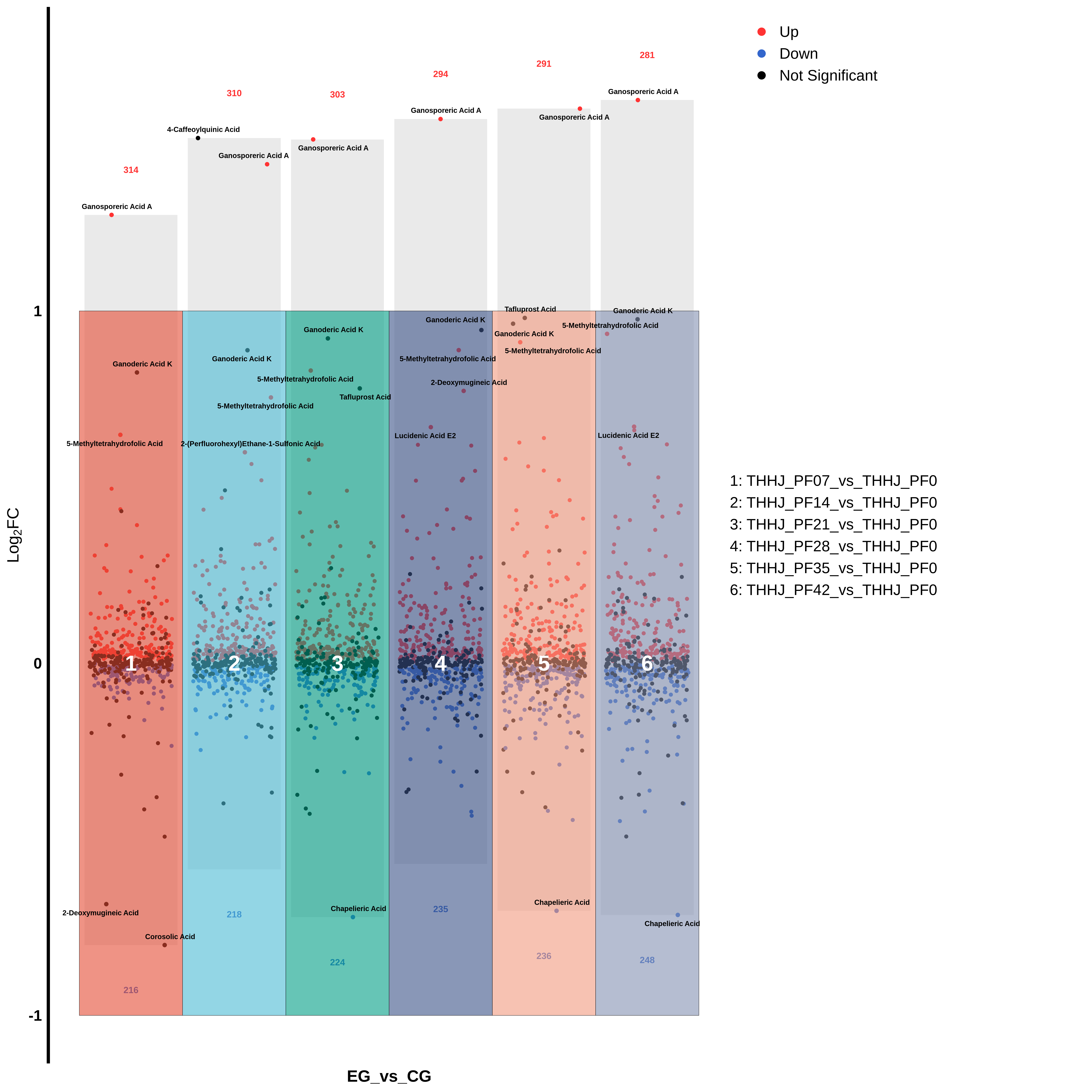

Supplement: Supplementary file 1 [file foods-15-00999-s001.zip › Figure S3(a)_Scatter plot of compounds bearing acid radicals (HMDB annotation) during post-fermentation process.png]

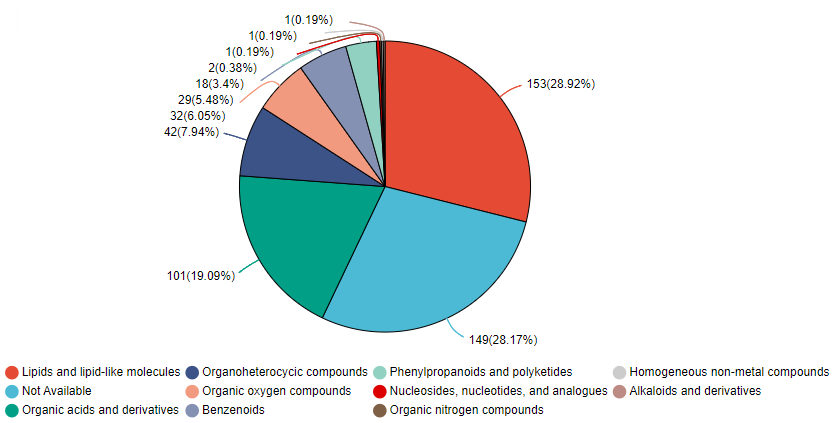

Supplement: Supplementary file 1 [file foods-15-00999-s001.zip › Figure S3(b)_Classification of compounds bearing acid radicals (HMDB super class).png]

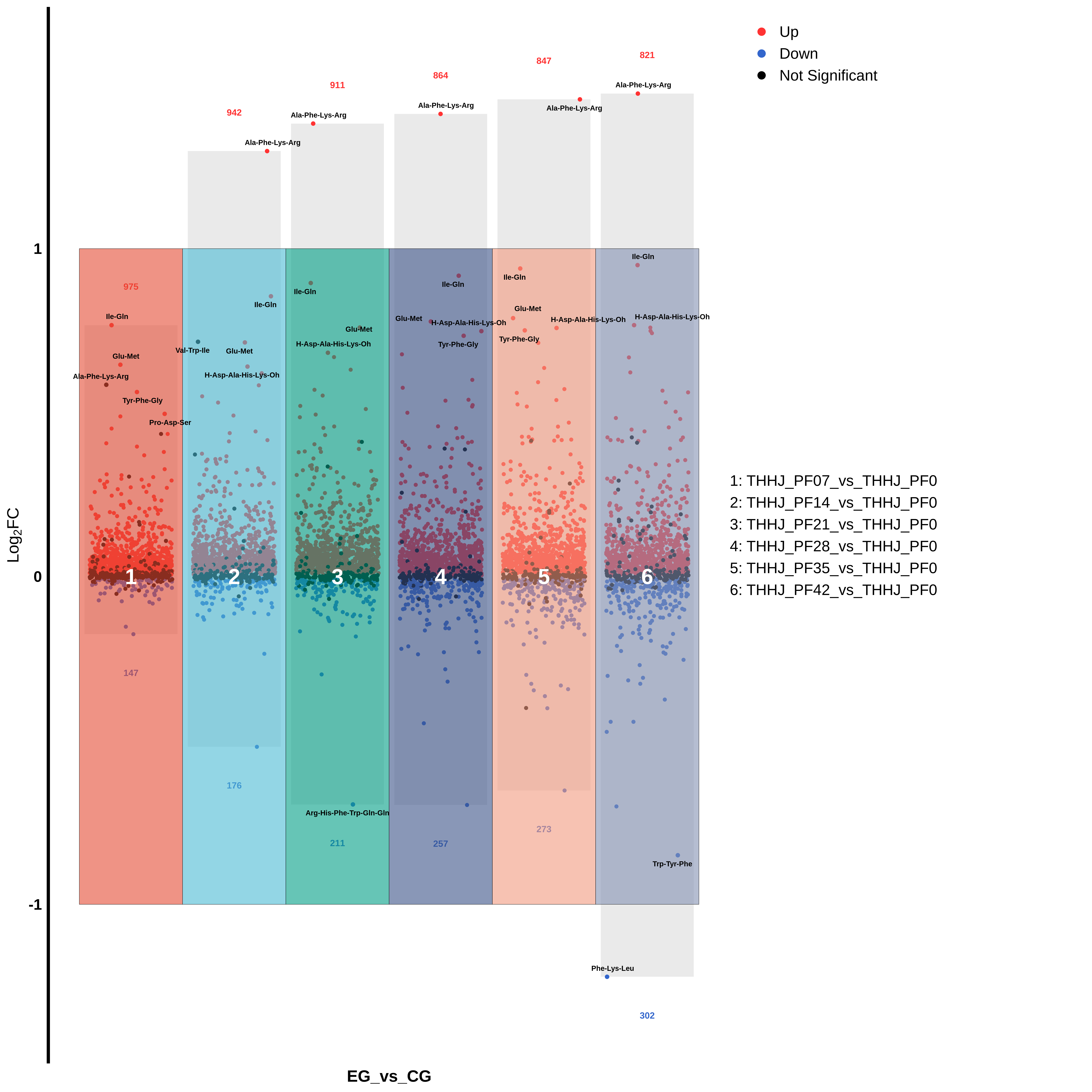

Supplement: Supplementary file 1 [file foods-15-00999-s001.zip › Figure S4(a)_Scatter plot of peptide during post-fermentation process.png]

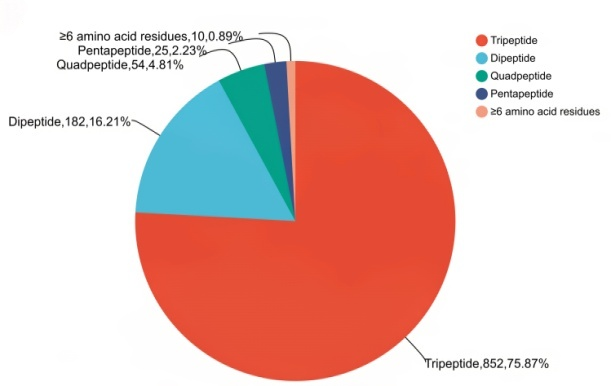

Supplement: Supplementary file 1 [file foods-15-00999-s001.zip › Figure S4(b)_Types of peptides.png]

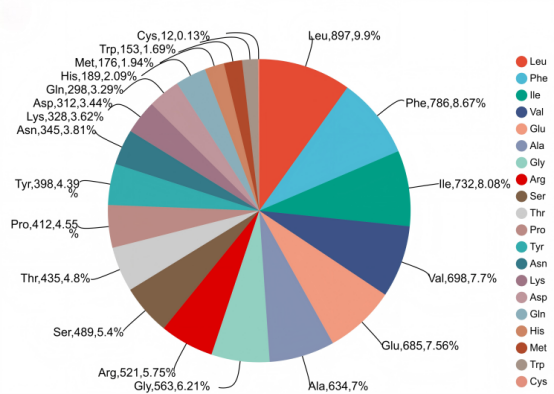

Supplement: Supplementary file 1 [file foods-15-00999-s001.zip › Figure S4(c)_Amino acid residues in peptides.png]
